# Identification of Effective Anticancer G-Quadruplex-Targeting Chemotypes through the Exploration of a High Diversity Library of Natural Compounds

**DOI:** 10.3390/pharmaceutics13101611

**Published:** 2021-10-03

**Authors:** Chiara Platella, Francesca Ghirga, Pasquale Zizza, Luca Pompili, Simona Marzano, Bruno Pagano, Deborah Quaglio, Valeria Vergine, Silvia Cammarone, Bruno Botta, Annamaria Biroccio, Mattia Mori, Daniela Montesarchio

**Affiliations:** 1 Department of Chemical Sciences, University of Naples Federico II (Complesso Universitario di Monte S. Angelo), Via Cintia, 21, 80126 Napoli, Italy; chiara.platella@unina.it; 2 Department of Chemistry and Technology of Drugs, “Department of Excellence 2018−2022”, Sapienza University of Rome, P.le Aldo Moro 5, 00185 Rome, Italy; francesca.ghirga@uniroma1.it (F.G.); deborah.quaglio@uniroma1.it (D.Q.); valeria.vergine@uniroma1.it (V.V.); silvia.cammarone@uniroma1.it (S.C.); bruno.botta@uniroma1.it (B.B.); 3 Oncogenomic and Epigenetic Unit, IRCCS—Regina Elena National Cancer Institute, Via Elio Chianesi 53, 00144 Rome, Italy; pasquale.zizza@ifo.gov.it (P.Z.); luca.pompili@ifo.gov.it (L.P.); annamaria.biroccio@ifo.gov.it (A.B.); 4 Department of Pharmacy, “Department of Excellence 2018−2022”, University of Naples Federico II, Via D.Montesano, 49, 80131 Napoli, Italy; simona.marzano@unina.it (S.M.); bruno.pagano@unina.it (B.P.); 5 Department of Biotechnology, Chemistry and Pharmacy, “Department of Excellence 2018-2022”, University of Siena, Via Aldo Moro 2, 53100 Siena, Italy

**Keywords:** G-quadruplex, natural compounds, chelidonine, rotenone, high diversity library, telomere, cancer, G4-CPG assay, molecular dynamics

## Abstract

In the quest for selective G-quadruplex (G4)-targeting chemotypes, natural compounds have been thus far poorly explored, though representing appealing candidates due to the high structural diversity of their scaffolds. In this regard, a unique high diversity in-house library composed of ca. one thousand individual natural products was investigated. The combination of molecular docking-based virtual screening and the G4-CPG experimental screening assay proved to be useful to quickly and effectively identify—out of many natural compounds—five hit binders of telomeric and oncogenic G4s, i.e., Bulbocapnine, Chelidonine, Ibogaine, Rotenone and Vomicine. Biophysical studies unambiguously demonstrated the selective interaction of these compounds with G4s compared to duplex DNA. The rationale behind the G4 selective recognition was suggested by molecular dynamics simulations. Indeed, the selected ligands proved to specifically interact with G4 structures due to peculiar interaction patterns, while they were unable to firmly bind to a DNA duplex. From biological assays, Chelidonine and Rotenone emerged as the most active compounds of the series against cancer cells, also showing good selectivity over normal cells. Notably, the anticancer activity correlated well with the ability of the two compounds to target telomeric G4s.

## 1. Introduction

Advanced technologies have allowed an in-depth understanding of the structural and biological features of G-quadruplex (G4) structures and evidence pointed to the biological relevance of G4 nucleic acids, particularly as targets in anticancer strategies [1,2,3,4]. To date, several synthetic compounds have been identified as selective ligands able to bind and stabilize G4 structures, with some of them showing effective anticancer activity in vivo and, therefore, being evaluated in advanced clinical trials [5,6,7,8,9]. Nevertheless, none of the most promising G4 ligands have been approved as a drug thus far. For the real progression of anticancer therapies based on G4-targeting ligands the investigation of large libraries of small molecules endowed with high chemical diversity is, therefore, strongly needed to identify novel chemotypes. In this context, natural compounds have been poorly studied as G4 ligands compared to synthetic compounds [5,10,11,12], though representing, in principle, appealing candidates due to the remarkable structural diversity of their scaffolds.

Plants are a rich source of structurally diverse secondary metabolites, which can be exploited in the development of new drug candidates [13,14]. Due to their high biodiversity, medicinal plants provide a huge number of natural compounds [15,16,17]. However, less than 1% of this biodiversity has been exploited in drug discovery due to several factors including the lack of a proper multidisciplinary view. The advent of powerful and efficient methods, such as the integrated combination of combinatorial chemistry and High-Throughput Screening (HTS), as well as user-friendly informatics tools, such as computer-aided drug design, which met the demand of major pharmaceutical companies to accelerate the research process, promoted the revolution of natural products screenings in drug discovery [18,19]. In addition, the development of new techniques for the isolation and characterization of novel compounds significantly improved the efficiency of the processes, in which the major challenges can currently be identified in the generation of high-quality libraries of diverse natural products that might allow the fast identification of lead compounds of pharmacological relevance [20,21,22].

A unique high diversity library composed of ca. one thousand individual natural products, isolated mainly from indigenous plants collected in biodiversity-rich countries, especially in tropical and subtropical areas, and enlarged with their semi-synthetic and synthetic derivatives, is available from the Organic Chemistry Laboratory of the Department of Chemistry and Technology of Drugs of Sapienza University of Rome, Italy [23]. During the years, the exploitation of this in-house collection of natural products offered a unique chance to identify unexpected new scaffolds for the development of therapeutically relevant molecules. Furthermore, the successful application of computer-aided methods in screening this unique and diverse in-house library provided some lead compounds that have been developed and, in some cases, patented as anticancer and antimicrobial agents [24,25,26,27,28]. Here, a docking-based virtual screening has been carried out, using both telomeric and oncogenic G4 models as targets [29,30], to evaluate the ability of the in-house natural products to target G4 grooves and identify novel G4-targeting chemotypes. Groove and loop binders are expected to be more selective than compounds that stack on top of the guanine quartets, although structural details of the highly flexible G4 loops are generally not precise enough to allow a reliable docking-based virtual screening in these regions compared to G-tetrads [31]. The virtual screening process identified 28 potential selective G4 ligands (Table S1) which, to the best of our knowledge, have not been previously investigated in their interaction with G4 structures, with the only exception of Aloin and Chelidonine for which only preliminary studies are reported in the literature [32,33]. Then, the ligands prioritized in silico have been experimentally screened by exploiting the G4-CPG (G-quadruplex on Controlled Pore Glass) assay, an affinity chromatography-based method to efficiently and quickly identify G4 selective ligands [11,34,35,36,37,38,39]. The compounds showing the highest affinity and selectivity for the selected G4 models have been studied for their interaction with the G4-forming sequences of choice in solution, in parallel with a duplex structure as control, by using circular dichroism (CD) and fluorescence spectroscopies. Additionally, molecular dynamics (MD) simulations and biological studies have been carried out for the best G4 ligands in order to get a deeper insight into their binding behavior towards G4 structures as well as their antiproliferative activity on cancer and normal cells.

## 2. Results and Discussion

### 2.1. Docking-Based Virtual Screening of the In-House Library of Natural Compounds

The unique high diversity in-house library investigated here consists of fully characterized natural products and their derivatives belonging to different classes of organic compounds, including variably substituted flavonoids, benzophenones, xanthones, anthraquinones, alkaloids, steroids, terpenoids etc. It was then enlarged with natural compounds from commercially available sources and semi-synthetic and synthetic compounds [23]. A docking-based virtual screening was carried out with the AutoDock program, using the solution structure of both a telomeric and an oncogenic G4 target as rigid receptors [29,30], and the solution structure of a DNA duplex as a negative control for the identification of potentially G4 selective virtual hits. As previously described [36], the binding site was centred on the G4s groove in the search for ligands that might be more selective than compounds that stack on top of the G4 tetrads. In detail, the rectangular binding site was centred in the groove formed by G4-G6, T8 and G22-T24 in tel_26_ and the groove formed by G2, A3, G5, G6 and G17-G19 in c-myc covering the groove and the loops. Although we are aware that this selection might not consider potential binders to other sites such as G-tetrad stackers, we believe that groove binders might be more profitable than unspecific stackers for further development. In contrast, the entire surface of the unspecific DNA duplex was scanned in the docking-based virtual screening. After docking, compounds were ranked based on their theoretical affinity within the groove of the G4 targets. The ligand-binding mode was visually inspected, and the compounds were further filtered by chemical diversity through a custom Python script for compounds clustering based on a combination of fingerprints and substructure search [40]. This latter step was aimed at reducing chemical redundancy within the test set and exploring the largest portion as possible of the natural products space represented in the in-house library. Specifically, the compound with the best score of each chemical cluster was selected for further processing. Finally, a comparison of the ligands scores in binding to the target G4s and the unspecific duplex sequence led to the final selection of 28 candidate hits (Table S1). A considerable part of these compounds belongs to the chemical class of alkaloids (**1**–**13**), one of the largest and most intriguing families of natural compounds. Indeed, alkaloids are characterized by vast structural diversity with no uniform classification. Several candidate hits belong to the chemical class of polyphenols (anthranoids, flavonoids and benzophenones). Within the anthranoids subclass, which can be chemically described as dihydroxy-anthraquinones, -dianthrones and -anthrones, three ferruginines (**14**, **15** and **16**), two anthrones (**17** and **18**) and one vismione (**19**) were selected. Among the flavonoids subclass, two rotenoids (**20** and **21**), containing a *cis*-fused tetrahydrochromeno [3,4-*b*]chromene nucleus, were identified. Among the polyphenols included in the library, a benzophenone (**22**) was identified. Three compounds were steroids (**23**, **24** and **25**), a subclass of terpenes featured by a characteristic molecular structure composed of 17 carbon atoms arranged in four rings. A Diels-Alder type adduct (**26**) and a naturally occurring dibenzofuran derivative (**27**), together with a cyanogenic glycoside (**28**), complete the test set.

### 2.2. Experimental Screening of the Library of 28 Natural Compounds by the G4-CPG Assay

The 28 natural compounds in silico selected as G4 ligands were experimentally evaluated for their ability to interact with G4 structures by the G4-CPG assay, an affinity chromatography-based method for the screening of putatively selective G4 ligands [11,34,35,36,37].

As in molecular docking, two cancer-related G4-forming DNA sequences originated from human telomeres (tel_26_) or c-myc oncogene promoter (c-myc) were used as the targets [29,41]. In parallel, the interaction with a model unimolecular duplex-forming DNA sequence (ds_27_) was also examined to determine if the analyzed compounds could discriminate G4 vs. duplex DNA structures [37].

The 28 compounds were dissolved in DMSO to prepare stock solutions. Then, they were evaluated for their solubility in the concentration and in the washing/releasing solutions used in the G4-CPG assay (see Materials and Methods for details). All tested compounds proved to be fully soluble and stable in the assay experimental conditions, except for Amygdalin, Clusiacitran B, Digitonin, Diosgenin, Solanidine and Usnic acid.

Successively, for each of the remaining 22 natural compounds, tests were carried out to evaluate, firstly the possible unspecific binding on the nude CPG support, and then their ability to bind G4- and duplex-forming DNA. The results of the G4-CPG assay are summarized in Table 1. Generally, no significant unspecific binding was observed, thus, not precluding further analysis on the oligonucleotide-functionalized supports. However, some unspecific binding on the nude CPG support was detected for Ferruanthrone, Ferruginin A, Ferruginin B, Hydrastine, Kuwanon G, Narceine and Rotenone. This was taken into due consideration in the following selection of the best G4 ligands.

Overall, 20-OH-Ecdysone, Aloin, Aspidospermine, Ferruginin B, γ,γ’-OH-Ferruginin A, Rotenolone, Veratrine, Vindoline, Vismione B and Yohimbine proved to weakly interact with G4- and duplex-functionalized supports. On the other hand, Emetine, Ferruanthrone, Ferruginin A, Hydrastine, Jervine and Narceine showed a good affinity for both G4- and duplex-functionalized supports, with no significant difference in terms of the percentage of bound ligand to the different secondary structures of the DNA investigated.

Conversely, Bulbocapnine, Chelidonine, Ibogaine, Kuwanon G, Rotenone and Vomicine showed a good affinity towards G4-functionalized supports and low-to-null interactions with the duplex-functionalized support, as also evidenced by the related indexes of G4/duplex selectivity (Table 1). However, Kuwanon G was not considered for further investigations due to its affinity towards nude CPG similar to tel_26_-functionalized CPG.

**Figure 1:**
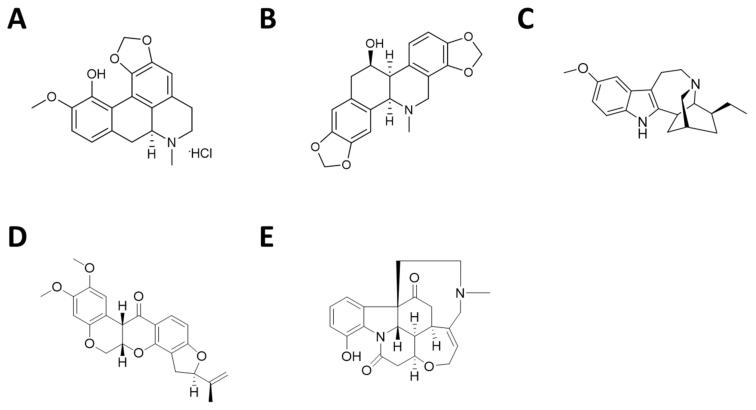
Chemical structures of (**A**) Bulbocapnine, (**B**) Chelidonine, (**C**) Ibogaine, (**D**) Rotenone and (**E**) Vomicine.

In summary, Bulbocapnine, Chelidonine, Ibogaine, Rotenone and Vomicine (Figure 1) were selected as the best G4 ligands in terms of affinity and selectivity over the DNA duplex, according to the results of the G4-CPG assay and were further analyzed in solution by biophysical techniques in order to achieve additional information on their interaction with G4 structures.

### 2.3. Circular Dichroism Studies

Considering the results of the G4-CPG assay, the ability of Bulbocapnine, Chelidonine, Ibogaine, Rotenone and Vomicine to interact with telomeric and oncogenic G4 models as well as a control duplex, was investigated in solution by CD experiments. All DNA oligonucleotides were prepared by overnight annealing tel_26_, c-myc and ds_27_ solutions at 2 μM DNA concentration, in 5 mM KCl, 5 mM phosphate buffer, 5% DMSO (pH 7). In full agreement with the literature data, in the above conditions, we found that: (i) tel_26_ folded into a hybrid G4, featured by a double hump-band, with maxima centered at 290 and 265 nm [42], (ii) c-myc adopted a parallel G4 topology, with a maximum centered at 262 nm and a minimum at 242 nm [42] and (iii) ds_27_ showed a positive band at 280 nm along with an intense minimum at 251 nm, characteristic of a B-DNA duplex structure (see Figures S1–S3, black lines) [43].

Then, the three secondary structure-forming oligonucleotides were titrated with increasing amounts of the five selected compounds (up to 10 molar equivalents), and the corresponding CD spectra were recorded after each addition (Figures S1–S3).

Bulbocapnine, Chelidonine, Ibogaine, Rotenone and Vomicine present one or more chiral centers. Aiming at evaluating the contribution of these ligands to the CD spectra obtained from titration experiments with the oligonucleotides, control CD spectra of these five compounds were recorded by adding increasing amounts of each ligand to the buffer alone, thus reproducing the above titration experiments but in the absence of DNA (Figure S4).

Thus, the contribution of each ligand was subtracted from the CD spectra obtained upon titrations of the tel_26_ and c-myc G4s or ds_27_ duplex, obtaining a more accurate picture of the spectral changes of the DNA oligonucleotides induced by each ligand (Figures S5–S7).

After these subtractions, it emerged that for the tel_26_/Ibogaine, tel_26_/Rotenone and tel_26_/Vomicine systems only slight spectral changes of tel_26_ G4 were detected (Figure S5C–E). On the other hand, relevant spectral changes were observed for tel_26_ G4 upon addition of Bulbocapnine or Chelidonine (Figure S5A,B).

For all the c-myc/ligand systems, a slight dose-dependent decrease of the CD intensity of the 262 nm band was observed (Figure S6). In addition, a slight dose-dependent increase of the CD intensity of the 242 nm band was found for the c-myc/Bulbocapnine and c-myc/Rotenone systems (Figure S6A,D).

As far as titrations of ds_27_ duplex are concerned, no relevant spectral changes were detected by inspection of the CD spectra after ligand contribution subtraction (Figure S7). Only slight spectral changes were found for the ds_27_/Bulbocapnine and ds_27_/Rotenone systems, with a dose-dependent decrease in CD signal intensity of the 280 nm band (Figure S7A,D).

**Figure 2:**
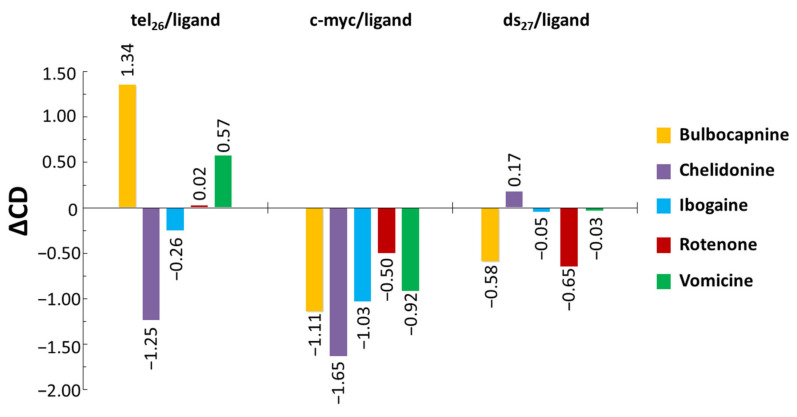
Bar graph representing ΔCD values calculated as the differences between the CD intensity of DNA/ligand 1:10 ratio systems (after ligand contribution subtraction) and the CD intensity of DNA alone considering the CD values at 290, 262 and 251 nm for tel_26_, c-myc and ds_27_ systems, respectively. The errors associated with ΔCD values are within ± 0.05.

To semi-quantitatively evaluate the spectral changes detected by titration of the tel_26_ and c-myc G4s or ds_27_ duplex, the differences (ΔCD) between the CD intensity of DNA/ligand 1:10 ratio systems (upon ligand contribution subtraction) and the CD intensity of DNA alone were calculated considering the CD values at 290, 262 and 251 nm for tel_26_, c-myc and ds_27_ systems, respectively. The obtained ΔCD values—to be intended as easy-to-handle parameters indicative of a trend, and not as quantitative data for the description of an observed effect—are reported in Figure 2.

In detail, the most significant effects on tel_26_ and c-myc G4 structures were induced by Bulbocapnine and Chelidonine, while the most relevant spectral changes on the ds_27_ duplex were detected for Bulbocapnine and Rotenone. Significantly, the highest ΔCD values for Bulbocapnine, Chelidonine, Ibogaine and Vomicine were found for the two investigated G4s compared to the control duplex structure, confirming their good G4 vs. duplex selectivity evidenced by the G4-CPG assay. On the other hand, Rotenone seemed to more markedly affect the duplex than the G4 structures. This finding is in line with its lower G4 vs. duplex selectivity compared to the other four compounds, as observed by the G4-CPG assay.

CD-melting experiments were also performed on all the DNA/ligand mixtures in the 5 mM KCl, 5 mM phosphate buffer, 5% DMSO (pH 7) to evaluate if stabilizing or destabilizing effects on the G4 and duplex structures were obtained upon incubation with each of the five ligands. CD-melting curves of tel_26_ and c-myc G4s or ds_27_ duplex in the absence or presence of each ligand (DNA/ligand 1:10 ratio) were recorded by following the CD changes at the wavelength of maximum intensity (290, 262 and 251 nm for tel_26_, c-myc and ds_27_, respectively) (Figure S8A–C). Melting temperatures for all the investigated systems are summarized in Table 2.

Moderate to low stabilizing effects on tel_26_ G4 were found for Chelidonine (ΔT_m_ = +4 °C), Ibogaine (ΔT_m_ = +2 °C), while no effect was detected for Bulbocapnine, Rotenone and Vomicine (Figure S8A and Table 2).

As concerns the c-myc systems, stabilizing effects were observed for all the investigated ligands. However, the T_m_ values could not be accurately determined for these systems due to the absence of good sigmoidal behavior of the related melting curves in the 10–95 °C range (Figure S8B). In order to overcome this drawback, melting experiments for the c-myc systems were also performed in a buffer containing a ten-fold lower potassium ions concentration, i.e., 0.5 mM KCl, 0.5 mM phosphate buffer, 5% DMSO (pH 7) (Figure S8D). Even at this very low K^+^ ion concentration, c-myc proved to fold in a G4, featured by parallel topology and a T_m_ of 45 °C (Figure S9). Under these conditions, melting temperatures were obtained for the c-myc/ligand systems showing a strong stabilizing ability of these compounds on c-myc G4 with ΔT_m_ = +13, +9, +5, +5 and +4 for Chelidonine, Bulbocapnine, Ibogaine, Vomicine and Rotenone, respectively (Figure S8D and Table 2).Notably, no or even destabilizing effects on ds_27_ duplex were observed for all the five compounds (Figure S8C and Table 2).

Overall, in full agreement with the G4-CPG assay and CD titrations, the results of the CD-melting experiments demonstrated that all the five investigated compounds showed an excellent ability to interact with G4s, also discriminating well the G4 vs. duplex structures.

### 2.4. Fluorescence Spectroscopy Studies

In order to get a deeper insight into the affinity of the selected natural compounds to the selected G4 and duplex DNA, fluorescence experiments were performed. First, the fluorescence behavior of the selected compounds was evaluated. In detail, fluorescence spectra of 2 μM solutions of each ligand were recorded in 5 mM KCl, 5 mM phosphate buffer, 5% DMSO (pH 7). Bulbocapnine, Chelidonine and Ibogaine showed strong emission bands at 460, 330 and 356 nm, respectively (Figures S10–S12, left panels, black lines). Conversely, Rotenone and Vomicine did not show appreciable fluorescence intensity, thus hampering further investigations on DNA binding by fluorescence spectroscopy.

Then, fluorescence titrations were carried out for Bulbocapnine, Chelidonine and Ibogaine (Figures S10–S12, left panels) at a fixed concentration of ligand (i.e., 2 μM), by adding increasing amounts of tel_26_ and c-myc G4s or ds_27_ duplex, previously annealed in 5 mM KCl, 5 mM phosphate buffer, 5% DMSO (pH 7). Upon each addition, the corresponding fluorescence spectrum was recorded after the stabilization of the signal. Successively, for each system, the fraction of bound ligand was calculated from the obtained fluorescence intensity values and plotted as a function of the DNA concentration (Figures S10–S12, right panels). These data were then fitted with an independent and equivalent-sites model [44] to calculate the binding constants (*K*_b_) (Table 3).

Titrations of Bulbocapnine with increasing amounts of tel_26_ and c-myc G4s or ds_27_ duplex showed an overall fluorescence quenching (Figure S10A–C). *K*_b_ values of 1.0, 1.1 and 1.2 × 10^6^ M^−1^ were obtained for Bulbocapnine binding to tel_26_, c-myc and ds_27_ systems, respectively (Figure S10D–F and Table 3).

As far as Chelidonine is concerned, an overall quenching effect was observed on increasing tel_26_ G4 concentration (Figure S11A). On the other hand, the c-myc/Chelidonine system (Figure S11B) exhibited an initial fluorescence enhancement (from 0 to 1 μM DNA concentration) followed by fluorescence quenching (from 1 to 10 μM DNA concentration). Notably, the fluorescence for the ds_27_/Chelidonine system did not significantly change by increasing DNA concentration (Figure S11C). Unfortunately, by plotting fluorescence intensity at the wavelength of Chelidonine intensity maximum vs. added DNA increasing concentration (Figure S11D–F), the experimental data did not follow a well-defined behavior, thus not allowing adopting fitting protocols to obtain binding constants.

As in the case of Bulbocapnine, a significant fluorescence quenching was observed upon titration of Ibogaine with all the investigated oligonucleotides (Figure S12A–C). *K*_b_ values of 4.1, 3.9 and 3.7 × 10^5^ M^−1^ were obtained for Ibogaine interaction with tel_26_, c-myc and ds_27,_ respectively (Figure S12D–F and Table 3).

Overall, while higher binding constants were found for Bulbocapnine with tel_26_ and c-myc G4s as well as ds_27_ duplex compared to Ibogaine, the latter one showed slightly higher G4 vs. duplex selectivity than Bulbocapnine. Notably, the presence or absence of significant fluorescence intensity variations for Chelidonine upon titration with the G4s or the duplex, respectively, further evidenced a good G4 vs. duplex selectivity for this compound, in agreement with G4-CPG assay and CD results.

### 2.5. Molecular Dynamics Simulations

The tandem application of in silico and experimental screenings proved very useful in identifying five hit binders of tel_26_ and c-myc G4 structures out of many natural compounds. However, the molecular docking-based virtual screening relied on the use of static receptor structures, solved in non-physiological conditions, which can fail to address loops flexibility of the G4 targets. Thus, to investigate the coherence and persistency of binding modes identified by molecular docking, MD simulations were run on all the examined G4/ligand complexes. In addition, MD simulations were run on duplex/ligand complexes to explore structural determinants for the observed selectivity for G4s. For each complex, MD trajectories were produced for 500 ns without positional restraints, and the ligands’ theoretical affinity was estimated by the Molecular Mechanics Generalized Born Surface Area (MM-GBSA) approach [45]. Finally, MD frames were clustered, and the most representative structure was used for further discussion and structural speculations.

**Figure 3:**
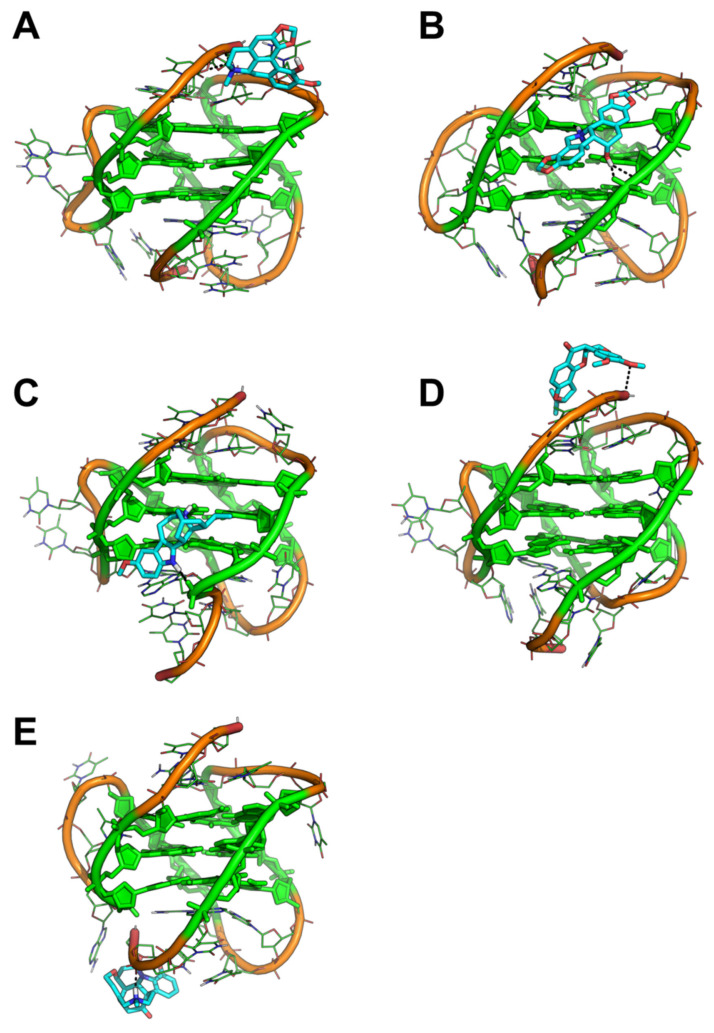
Representative binding conformation of selected ligands to tel_26_ G4 extracted from MD trajectories. (**A**) tel_26_ G4/Bulbocapnine; (**B**) tel_26_ G4/Chelidonine; (**C**) tel_26_ G4/Ibogaine; (**D**) tel_26_ G4/Rotenone; (**E**) tel_26_ G4/Vomicine. The G4 is shown as lines and cartoon. G nucleotides forming G-quartets are shown as green sticks with filled rings. Small molecules are shown as cyan sticks. Metal ions and water molecules have been omitted for the sake of clarity. tel_26_ G4/ligand polar interactions are highlighted by black dashed lines. The same orientation of each tel_26_ G4/ligand complex is shown, which was obtained by structural alignment on the G4 phosphate backbone.

In binding to tel_26_ G4, compounds showed a peculiar behavior (Figure 3). Chelidonine and Ibogaine preserved the binding site identified by molecular docking within the G4 groove and established H-bond interactions with the phosphate oxygen of G residues from G-quartets (Figure 3B,C). Bulbocapnine moved towards the 3′-end of the DNA strand and established an H-bond with the OH oxygen of 3′-T, although a strong stabilization seems to be determined by stacking interactions with a T from the edgewise (or lateral) loop (Figure 3A). Similarly, Rotenone and Vomicine detached from the groove to find their preferred binding location in a region on top of the G-quartets characterized by sequence specificity. Specifically, thanks to its L-shape, Rotenone was H-bonded to the OH oxygen of 3′-T as well as stacked on the same nucleobase (Figure 3D), while Vomicine bound within the 5′-end of the sequence by H-bonding the phosphate oxygen of T at position 2 and being stacked by 5′-T and an A from the propeller loop (Figure 3E).

**Figure 4:**
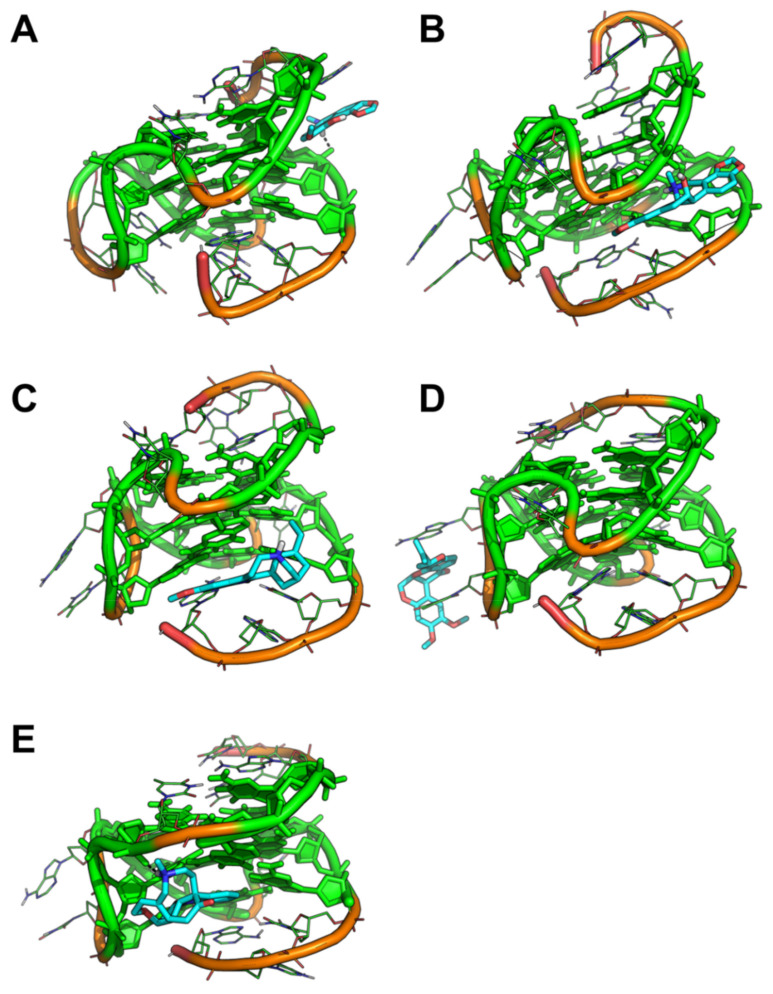
Representative binding conformation of selected ligands to c-myc G4 extracted from MD trajectories. (**A**) c-myc G4/Bulbocapnine; (**B**) c-myc G4/Chelidonine; (**C**) c-myc G4/Ibogaine; (**D**) c-myc G4/Rotenone; (**E**) c-myc G4/Vomicine. The G4 is shown as lines and cartoon. G nucleotides forming G-quartets are shown as green sticks with filled rings. Small molecules are shown as cyan sticks. Metal ions and water molecules have been omitted for the sake of clarity. c-myc G4/ligand polar interactions are highlighted by black dashed lines. The same orientation of each c-myc G4/ligand complex is shown, which was obtained by structural alignment on the G4 phosphate backbone.

In contrast to tel_26_ systems, MD simulations on the complexes between selected ligands and c-myc provided conformational results that were more coherent with docking outcomes. Indeed, Bulbocapnine, Chelidonine and Ibogaine stably bound within the G4 grooves (Figure 4A–C). Bulbocapnine and Chelidonine established H-bond interactions with the phosphate backbone. In contrast, Rotenone detached from the groove and moved to the propeller loop where it was sandwiched between the A and T of the loop, highlighting the propensity of this compound to establish aromatic interactions with single-stranded oligonucleotide tracts (Figure 4D). Vomicine moved slightly towards the 3′-end of the c-myc sequence where it bound to the terminal part of the groove being stacked onto a G from the G-tetrad as well as H-bonded to the phosphate backbone (Figure 4E).

**Figure 5:**
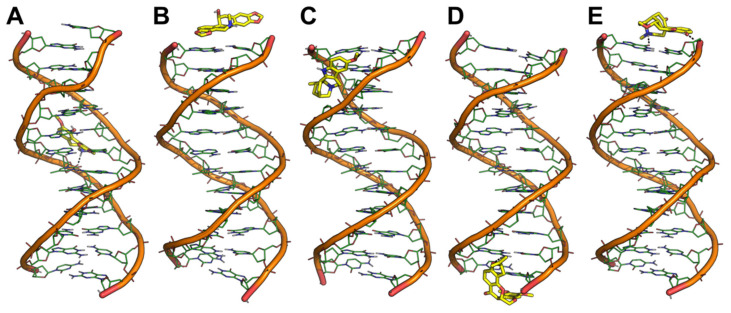
Representative binding conformation of selected ligands to a DNA duplex extracted from MD trajectories. (**A**) duplex/Bulbocapnine; (**B**) duplex/Chelidonine; (**C**) duplex/Ibogaine; (**D**) duplex/Rotenone; (**E**) duplex/Vomicine. The DNA duplex is shown as lines and cartoon. Small molecules are shown as yellow sticks. Metal ions and water molecules have been omitted for the sake of clarity. Duplex/ligand polar interactions are highlighted by black dashed lines. The same orientation of each duplex/ligand complex is shown, which was obtained by structural alignment on the phosphate backbone.

MD simulations were also run on the docking complexes between selected ligands and the DNA duplex model, to provide a rational structural explanation of the G4 vs. duplex selectivity observed by the G4-CPG assay, as well as by CD and fluorescence studies. Notably, only Bulbocapnine was found to be firmly bound to the duplex groove in correspondence of an AT-rich sequence, establishing an H-bond with the phosphate backbone (Figure 5A). All other compounds moved to the terminal ends of the duplex, where they bound non-specifically to the terminal base pair (Figure 5B–E). Since, in living cells, the DNA duplex does not have frequent chain breaks, this behavior might be interpreted as a weak affinity of the ligands for this nucleic acid structure. Only Rotenone was able to perform a rather specific interaction with the terminal end of the duplex, consisting of a π-stacking with 3′-G of a strand and an H-bond with G at position 2 of the opposite strand (Figure 5D).

Theoretical affinity of ligands to nucleic acid sequences tel_26_ and c-myc was estimated by the MM-GBSA approach along with the most populated cluster of MD frames. Theoretical affinity to the DNA duplex was not reported, as the compounds were bound in a pose that is not consistent with their possible behavior in a physiological context. Results were reported in Table 4 and remarkably highlight an affinity scenario that is highly comparable to that observed by CD and fluorescence spectroscopy. Overall, theoretical affinity data suggest a stronger binding to c-myc compared to tel_26_ G4, as highlighted by CD and thermal melting experiments, as well as a tighter affinity for Bulbocapnine and Chelidonine followed by Ibogaine and Vomicine, whereas Rotenone is the weakest binder of the series in agreement with experimental results.

Taken together, results of MD simulations suggest that selected ligands bind specifically to tel_26_ and c-myc G4 structures with a peculiar interaction pattern, being unable to bind the groove of a DNA duplex model, except for Bulbocapnine. The agreement between computational and experimental trends corroborates the predicted binding mode and sheds further light on the mechanism of action of these natural modulators of tel_26_ and c-myc G4s.

### 2.6. Evaluation of Biological Activity of the Identified G4 Ligands

**Figure 6:**
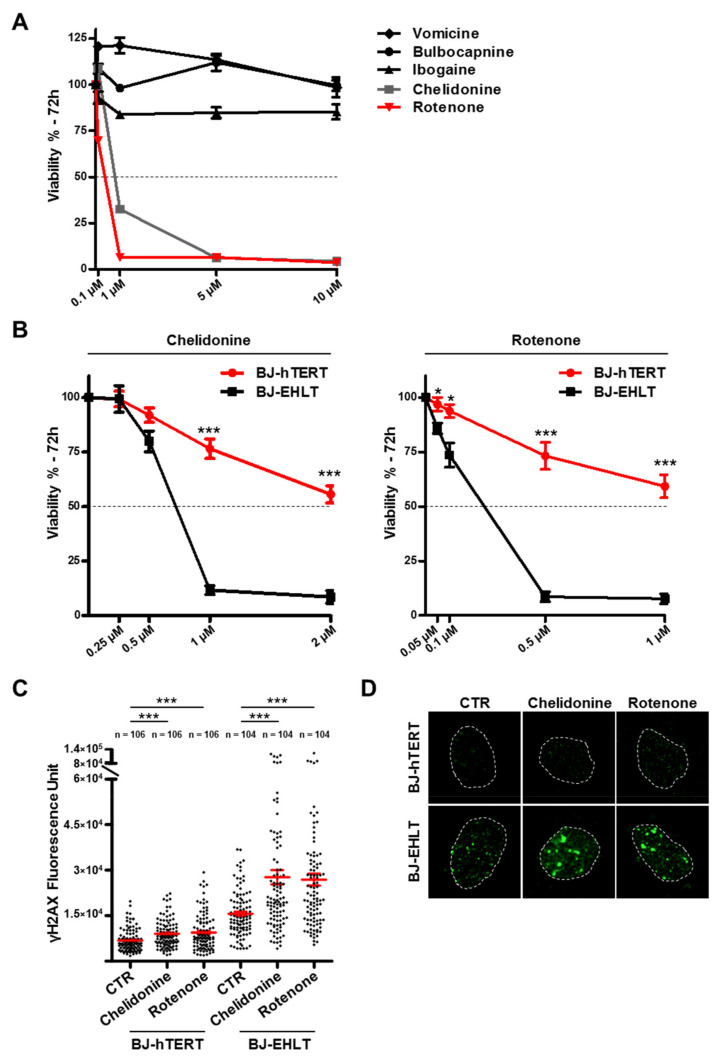
Chelidonine and Rotenone exhibit potential and selective anti-tumor activity. Data of viability and immunofluorescence assays. (**A**) Viability screening of candidate molecules. (**B**) Selective activity of Chelidonine and Rotenone against transformed fibroblast BJ-EHLT. (**C**) Quantitative analysis of fluorescence intensity of γH2AX signal in BJ-hTERT and BJ-EHLT. (**D**) Representative images of γH2AX fluorescent signal. ns, *p* > 0.05; *, *p* < 0.05; ***, *p* < 0.001.

Starting from the results of the biophysical and in silico studies, we proceeded to explore the biological activity of the identified G4 ligands (Bulbocapnine, Chelidonine, Ibogaine, Rotenone and Vomicine). To this aim, the antitumor potential of the candidate molecules was firstly assessed. In detail, BJ-EHLT cells, a line of human transformed fibroblasts, were treated with growing doses (from 0.1 to 10 µM for 72 h) of each of the five compounds and cell viability was evaluated by crystal violet assay. Notably, while Bulbocapnine, Ibogaine and Vomicine were almost ineffective, Chelidonine and Rotenone were found to produce a dose-dependent effect on cell viability (Figure 6A), with an estimated IC50 of 0.64 µM and 0.15 µM, respectively. These results prompted a specific focus on the biological characterization of the latter two compounds.

Thus, the selectivity of these biologically active compounds against malignant cells was then tested. To address this point, BJ-EHLT cells and their normal counterpart, BJ-hTERT, were exposed to treatment with Chelidonine and Rotenone for 72 h at the indicated doses, and the effect on cell viability was evaluated (Figure 6B). Interestingly, the growth curves, besides confirming the efficacy of the two treatments on transformed fibroblasts, showed very poor activity of both the ligands on normal cells, providing evidence of the selectivity of these molecules against tumor cells. Moreover, considering that anti-tumor activity of G4 ligands mainly depends on their capability to induce DNA damage [46], BJ-EHLT and BJ-hTERT were treated for 24 h with 1 µM of either Chelidonine or Rotenone and the amount of phosphorylated histone H2AX (γH2AX), a typical hallmark of DNA double-strand breaks [47], was estimated by immunofluorescence (IF) microscopy. Quantitative analysis of fluorescence intensity of γH2AX signal, evaluated on at least 100 nuclei (Figure 6C), showed that both the ligands are more effective in transformed than in normal cells (Figure 6D). Notably, for both treatments, the mean of the γH2AX signal in BJ-EHLT was three times higher than in BJ-hTERT, with an increase in the signal that in BJ-hTERT was approximately 30% compared to untreated cells (CTR) but reached 75% in BJ-EHLT. These results suggested that the effect of the G4 ligands on cell viability could be due to the capability of these compounds to induce selective DNA damage in transformed cells.

**Figure 7:**
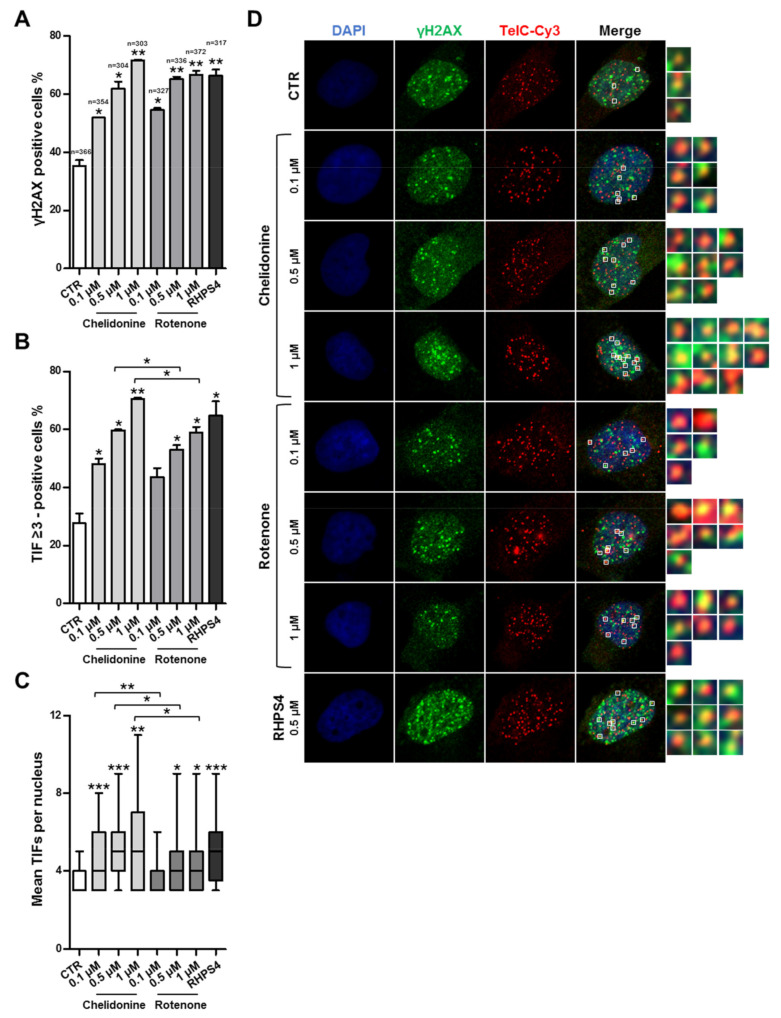
Chelidonine and Rotenone induce telomeric DNA damage. Quantitative analyses of data obtained by FISH. (**A**) Dose-dependent increase in γH2AX-positive cells. (**B**) Percentage increase in co-localizations between γH2AX and telomeric probe (TIFs) per nucleus. (**C**) Mean number of TIFs per nucleus. (**D**) Representative immunofluorescence microscopy images. Enlargements of co-localization spots (in yellow). ns, *p* > 0.05; *, *p* < 0.05; **, *p* < 0.01; ***, *p* < 0.001.

Additionally, to assess the ability of the two biologically active compounds to target G4 structures in cells, a fluorescence in situ hybridization (FISH) assay was performed. The analysis of telomeric damage, evaluated by quantification of the co-localization spots of γH2AX with a fluorescent telomeric probe (Telomere Induced Foci, TIF), confirmed, on one hand, the capability of Chelidonine and Rotenone to elicit DNA damage in a dose-dependent manner (Figure 7A,D) and, on the other hand, demonstrated that a large part of this damage was telomere-located (Figure 7B,D). Interestingly, both compounds exhibited a capability of inducing TIFs similar to pentacyclic acridine derivative RHPS4, a well-known G4 ligand used here as a positive control [48]. In particular, Chelidonine appeared as the best of the two ligands, reaching the highest percentage of TIF-positive cells with a mean number of five TIFs per nucleus (Figure 7C,D).

**Figure 8:**
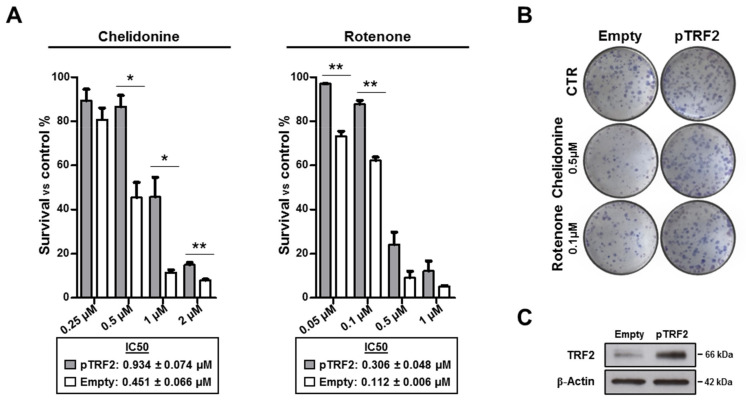
TRF2 over-expression protects cells from effect of Chelidonine and Rotenone. Clonogenic assay on MDA-MB-231 stably over-expressing TRF2. (**A**) Dose-dependent colony formation decrease upon Chelidonine and Rotenone treatment. (**B**) Representative images of colony survival. (**C**) Representative Western blot of TRF2 over-expression. ns, *p* > 0.05; *, *p* < 0.05; **, *p* < 0.01.

Finally, the viability assays were extended to a human breast cancer cell line (MDA-MB-231; Figure 8A,B) over-expressing TRF2 (pTRF2; Figure 8C), a telomeric protein playing a key role in telomere protection [49,50]. Here, TRF2 over-expression was used as a tool aimed at definitively proving that the anti-tumor activity of Chelidonine and Rotenone was related to telomere targeting. Interestingly, under TRF2 over-expression, cells were preserved from the cytotoxic effect of the two compounds, as demonstrated by IC50 values that increased from 0.451 ± 0.066 to 0.934 ± 0.074 µM for Chelidonine and from 0.112 ± 0.006 to 0.306 ± 0.048 µM for Rotenone.

Altogether, our biological data led to the identification of two natural compounds that exhibited a potent DNA damage-mediated cytotoxic activity, selectively targeting telomeric G4s. Notably, between the two biologically active compounds, the highest selectivity of action on cancer over normal cells along with the highest specificity in telomere targeting and damaging was found for Chelidonine, in full agreement with its higher selectivity for G4 over duplex DNA than Rotenone, as determined by the G4-CPG assay and the CD-melting data.

## 3. Conclusions

Aiming at searching selective G4 ligands as putative candidate drugs for anticancer targeted therapies, a unique high-diversity library of natural compounds has been investigated. A molecular docking-based virtual screening identified 28 putative G4 ligands that were then evaluated by the G4-CPG experimental screening assay, whereby five molecules were confirmed as effective G4 ligands, i.e., Bulbocapnine, Chelidonine, Ibogaine, Rotenone and Vomicine. Then, CD and fluorescence spectroscopy indicated that the five investigated compounds can interact with G4s, also selectively stabilizing the G4 vs. duplex structures.

A detailed inspection of biophysical data revealed that the highest stabilizing effects and affinity on G4 over duplex structures were detected for Chelidonine, while the lowest ones were observed for Rotenone, in line with its lower G4 vs. duplex selectivity compared to the other four compounds as observed by the G4-CPG assay. For Chelidonine a stable binding to grooves of G4 structures was suggested by MD simulations along with its inability to firmly bind to duplex DNA, while Rotenone was proven to mainly target G4 flanking or loop residues, as well as to perform a rather specific interaction with the terminal end of the duplex DNA.

Moreover, Chelidonine and Rotenone, whose anti-tumor potential has been evaluated also in recent reports [51,52], were found to produce a potent anticancer activity mediated by their capability to bind and stabilize telomeric G4 structures. In particular, demonstrating that both compounds are effective at low µM doses and show selectivity for tumor cells, our results pave the way for the design of novel and even more effective synthetic analogs of these natural compounds that could find their application field in anticancer therapies. In this regard, NMR studies on the interaction of the selected natural compounds with G4 models are currently underway in our labs aiming at obtaining high-resolution structures of their complexes with G4s and conclusively establishing their ability to target the G4 grooves/loops.

## 4. Materials and Methods

### 4.1. Chemistry

All the tested compounds (**1**–**28**) are known structures belonging to the in-house library of natural products available from the Organic Chemistry Laboratory of the Department of Chemistry and Technology of Drugs of Sapienza University of Rome, Italy. The chemical identity of compounds was assessed by re-running NMR experiments and proved to be in agreement with the literature data reported below for each compound. The purity of all compounds, checked by reversed-phase High-Performance Liquid Chromatography (HPLC), was always higher than 95%.

Compound **1** (Bulbocapnine hydrochloride or (S)-11-methoxy-7-methyl-6,7,7a,8-tetrahydro-5H-[1,3]dioxolo[4’,5′:4,5]benzo[1,2,3-de]benzo[g]quinolin-12-ol hydrochloride) was purchased from Sigma-Aldrich (CAS: 632-47-3, St. Louis, MO, USA) and used without further purification.

Compound **2** (Chelidonine or (5bR,6S,12bS)-13-Methyl-5b,6,7,12b,13,14-hexahydro[1,3]dioxolo[4′,5′:4,5]benzo[1,2-c][1,3]dioxolo[4,5-i]phenanthridin-6-ol) was purchased from Sigma-Aldrich (CAS: 476-32-4, St. Louis, MO, USA) and used without further purification.

Compound **3** (Emetine hydrochloride or (2S,3R,11bS)-2-(((R)-6, 7-dimethoxy-1,2,3,4- tetrahydroisoquinolin-1-yl)methyl)-3-ethyl-9,10-dimethoxy-2,3,4,6,7,11b-hexahydro-1H-pyrido[2,1-a] isoquinoline hydrochloride) was purchased from MolPort (CAS: 14198-59-5, Beacon, NY, USA) and used without further purification.

Compound **4** (Hydrastine or (R)-6,7-dimethoxy-3-((R)-6-methyl-5,6,7,8-tetrahydro-[1,3]dioxolo[4,5-g]isoquinolin-5-yl)isobenzofuran-1(3H)-one) was purchased from Sigma-Aldrich (CAS: 118-08-1, St. Louis, Mo., USA) and used without further purification.

Compound **5** (Narceine or 6-[2-[6-[2-(dimethylamino)ethyl]-4-methoxy-1,3-benzodioxol-5-yl]acetyl]-2,3-dimethoxybenzoic acid) was purchased from Sigma-Aldrich (CAS: 131-28-2, St. Louis, MO, USA) and used without further purification.

Compound **6** (Aspidospermine or 1-((3aR,5aR,10bR,12bR)-3a-Ethyl-7-methoxy-2,3,3a,5,5a,11,12,12b-octahydro-1H,4H-6,12a-diaza-indeno[7,1-cd]fluoren-6-yl)-ethanone) showed NMR spectra identical to those reported in the literature [53].

Compound **7** (Vomicine or (4aR,4a1R,6aS,6a1S,13aS)-10-hydroxy-16-methyl-4a,5, 13,13a-tetrahydro-2H-6a,4-(ethanoiminomethano)indolo[3,2,1-ij]oxepino[2,3,4-de]quinoline-6,12(4a1H, 6a1H)-dione) was purchased from MolPort (5969-84-6, Beacon, NY, USA) and used without further purification.

Compound **8** (Ibogaine or (6R,7S,11S)-7-ethyl-2-methoxy-6,6a,7,8,9,10,12,13-octahydro-5H-6,9- methanopyrido[1′,2′:1,2]azepino[4,5-b]indole) showed NMR spectra identical to those reported in the literature [54].

Compound **9** (Yohimbine hydrochloride or (1R,2S,4aR,13bS,14aS)-methyl 2-hydroxy-1,2,3,4,4a, 5,7,8,13,13b,14,14a-dodecahydroindolo[2′,3′:3,4]pyrido[1,2-b]isoquinoline-1-carboxylate hydrochloride) was purchased from Sigma-Aldrich (CAS: 65-19-0, St. Louis, MO, USA) and used without further purification.

Compound **10** (Jervine or (3β,23β)-17,23-Epoxy-3-hydroxyveratraman-11-one,11-Ketocyclopamine) was purchased from Sigma-Aldrich (CAS: 469-59-0, St. Louis, MO, USA) and used without further purification.

Compound **11** (Solanidine or (1S,2S,7S,10R,11S,14S,15R,16S,17R,20S,23S)-10,14,16,20-tetramethyl-22-azahexacyclo[12.10.0.02,11.05,10.015,23.017,22]tetracos-4-en-7-ol) was purchased from Sigma-Aldrich (CAS: 80-78-4,St. Louis, MO, USA) and used without further purification.

Compound **12** (Vindoline or (3aR,3a1R,4R,5S,5aR,10bR)-methyl 4-acetoxy-3a-ethyl-5-hydroxy-8-methoxy-6-methyl-3a,3a1,4,5,5a,6,11,12-octahydro-1H-indolizino[8,1-cd]carbazole-5-carboxylate) was purchased from MolPort (CAS: 2182-14-1, Beacon, NY, USA) and used without further purification.

Compound **13** (Veratrine hydrochloride or [(1*R*,2*S*,6*S*,9*S*,10*R*,11*S*,12*S*,14*R*,15*S*,18*S*,19*S*,22*S*,23*S*,25*R*)-1,10,11,12,14,23-hexahydroxy-6,10,19-trimethyl-24-oxa-4 azaheptacyclo[12.12.0.0^2,11^.0^4,9^.0^15,25^.0^18,23^.0^19,25^]hexacosan-22-yl] (*Z*)-2-methylbut-2-enoate) hydrochloride showed NMR spectra identical to those reported in the literature [55].

Compound **14** (Ferruginin A or 4,5,10-trihydroxy-7-methyl-1,1,6-tris(3-methylbut-2-enyl)anthracen-2-one) showed NMR spectra identical to those reported in the literature [56].

Compound **15** (γ,γ’-OH-Ferruginin A or 3,8,9-trihydroxy-4-(4-hydroxy-3-(hydroxymethyl)but-2-en-1-yl)-6-methyl-4,7-bis(3-methylbut-2-en-1-yl)anthracen-1(4H)-one) showed NMR spectra identical to those reported in the literature [57].

Compound **16** (Ferruginin B or 3,8,9-trihydroxy-6-methyl-2,4,4-tris(3-methylbut-2-en-1-yl)anthracen-1(4H)-one) showed NMR spectra identical to those reported in the literature [58].

Compound **17** (Ferruanthrone or 1,6,8-trihydroxy-3-methyl-2,4,7-tris(3-methylbut-2-en-1-yl)anthracen-9(10H)-one) showed NMR spectra identical to those reported in the literature [56].

Compound **18** (Aloin or 1,8-dihydroxy-3-(hydroxymethyl)-10-(3,4,5-trihydroxy-6-(hydroxymethyl)tetrahydro-2H-pyran-2-yl)anthracen-9(10H)-one) showed NMR spectra identical to those reported in the literature [59].

Compound **19** (Vismione B or 9,12-dihydroxy-5-methoxy-2,2,9-trimethyl-2,8,9,10-tetrahydro-11H-naphtho[2,3-h]chromen-11-one) showed NMR spectra identical to those reported in the literature [60].

Compound **20** (Rotenone or (2R,6aS,12aS)-8,9-dimethoxy-2-(prop-1-en-2-yl)-1,2,12,12a-tetrahydrochromeno[3,4-b]furo[2,3-h]chromen-6(6aH)-one) was purchased from TCI (Tokyo Chemical Industry) (CAS: 83-79-4, Tokyo, Japan) and used without further purification.

Compound **21** (Rotenolone or (2R,6S,6aR,12aS)-8,9-dimethoxy-2-(prop-1-en-2-yl)-1,2,6,6a,12,12a-hexahydrochromeno[3,4-b]furo[2,3-h]chromen-6-ol) showed NMR spectra identical to those reported in the literature [61].

Compound **22** (Clusiacitran B or (3-hydroxy-6,6,9-trimethyl-6a,7,8,9,10,10a-hexahydro-6H-1,9-epoxybenzo[c]chromen-2-yl)(phenyl)methanone) showed NMR spectra identical to those reported in the literature [62].

Compound **23** (Diosgenin or (4S,5’R,6aR,6bS,8aS,8bR,9S,10R,11aS,12aS,12bS)-5’,6a,8a,9-tetramethyl-1,3,3’,4,4’,5,5’,6,6a,6b,6’,7,8,8a,8b,9,11a,12,12a,12b-icosahydrospiro[naphtho[2’,1’:4,5]indeno[2,1-b]furan-10,2’-pyran]-4-ol) showed NMR spectra identical to those reported in the literature [63].

Compound **24** (Digitonin or (2*S*,3*R*,4*S*,5*S*,6*R*)-2-[(2*S*,3*R*,4*S*,5*S*,6*R*)-2-[(2*S*,3*R*,4*S*,5*R*,6*R*)-2-[(2*R*,3*R*,4*R*,5*R*,6*R*)-6-[(1*R*,2*S*,3*S*,4*R*,5’*R*,6*R*,7*S*,8*R*,9*S*,12*S*,13*S*,15*R*,16*R*,18*S*)-3,15-dihydroxy-5’,7,9,13-tetramethylspiro[5-oxapentacyclo[10.8.0.0^2,9^.0^4,8^.0^13,18^]icosane-6,2’-oxane]-16-yl]oxy-4,5-dihydroxy-2-(hydroxymethyl)oxan-3-yl]oxy-5-hydroxy-6-(hydroxymethyl)-4-[(2*S*,3*R*,4*S*,5*R*)-3,4,5-trihydroxyoxan-2-yl]oxyoxan-3-yl]oxy-3,5-dihydroxy-6-(hydroxymethyl)oxan-4-yl]oxy-6-(hydroxymethyl)oxane-3,4,5-triol)) was purchased from Sigma-Aldrich (CAS: 11024-24-1St. Louis, Mo., USA) and used without further purification.

Compound **25** (20-OH-Ecdysone or (2S,3R,5R,9R,10R,13R,14S,17S)-2,3,14-trihydroxy-10,13-dimethyl-17-((2R,3R)-2,3,6-trihydroxy-6-methylheptan-2-yl)-1,2,3,4,5,9,10,11,12,13,14,15,16,17-tetradecahydro-6H-cyclopenta[a]phenanthren-6-one) showed NMR spectra identical to those reported in the literature [64].

Compound **26** (Kuwanon G or 8-((1S,2R,3R)-2-(2,4-dihydroxybenzoyl)-2’,4’-dihydroxy-5-methyl-1,2,3,6-tetrahydro-[1,1’-biphenyl]-3-yl)-2-(2,4-dihydroxyphenyl)-5,7-dihydroxy-3-(3-methylbut-2-en-1-yl)-4H-chromen-4-one) showed NMR spectra identical to those reported in the literature [65].

Compound **27** (Usnic acid or (R)-1,1’-(1,7,9-trihydroxy-8,9b-dimethyl-3-oxo-3,9b-dihydrodibenzo[b,d]furan-2,6-diyl)bis(ethan-1-one) showed NMR spectra identical to those reported in the literature [66].

Compound **28** (Amygdalin or (2R)-2-phenyl-2-((2,3,4-trihydroxy-5-(((3,4,5-trihydroxy-6-(hydroxymethyl)tetrahydro-2H-pyran-2-yl)oxy)methyl)cyclohexyl)oxy)acetonitrile) showed NMR spectra identical to those reported in the literature [67].

### 4.2. Molecular Docking

The NMR structures of tel_26_ and c-myc G4s and a 12-mer DNA duplex were retrieved from the Protein Data Bank [68] under the accession code 2JPZ, 1XAV and 1NAJ, respectively [29,30,69]. The first NMR model was extracted from PDBs and used as a rigid receptor in molecular docking simulations carried out with the AutoDock4.2 program [70]. According to previous studies, the ligand-binding site was centered in the G4s groove [36]. Specifically, in the first NMR model of the tel_26_ structure, the binding site was centered in the groove formed by G4-G6, T8 and G22-T24 having 50 × 50 × 50 points dimension with a point-spacing of 0.375 Å. In the first NMR model of the c-myc structure, the ligand-binding site was centred on the loop formed by G2, A3, G5, G6, and G17-G19 having 60 × 60 × 60 points dimension with a point-spacing of 0.375 Å. In the first NMR model of the 12-mer DNA duplex, the binding site was centered in the mass center of A6-T19 and T7-A18 base pairs with a dimension of 60 × 100 × 60 points and a point-spacing of 0.375 Å to cover the entire surface of the unspecific duplex. Default AutoDock4.2 settings were used, and ten docking poses of each compound were stored. Ligands chemical diversity was evaluated by a Python-based clustering algorithm based on a combination of fingerprints and maximum common substructure search [40,71].

### 4.3. G4-CPG Assay

Nude CPG, tel_26_-, c-myc- and ds_27_-functionalized CPG supports were synthesized as previously reported [11,34,35,36,37]. The stock solutions for each ligand were prepared by dissolving a known amount of the sample in pure DMSO, thus, obtaining 4 mM solutions (with the only exception of Bulbocapnine used as a 3.3 mM solution). A measured volume was taken from the stock solution to obtain a 600 μM ligand solution in a 50 mM KCl, 10% DMSO, 10% CH_3_CH_2_OH aq. solution. The detailed general procedure adopted for the assays is described as follows: weighed amounts of nude CPG or G4- and duplex-functionalized CPG supports were left in contact with 300 μL of the 600 μM ligand solution in a polypropylene column equipped with a polytetrafluoroethylene frit, a stopcock and a cap. After incubation on a vibrating shaker for 4 min, each support was washed with defined volumes of the washing solution (50 mM KCl, 10% DMSO, 10% CH_3_CH_2_OH aq. solution) or the releasing solution (2.5 M CaCl_2_, 15% DMSO aq. solution or pure DMSO) and all the eluted fractions were separately analyzed by UV measurements. After treatment with the releasing solution, inducing G4s and duplex denaturation, the G4- and duplex-functionalized CPG supports were suspended in the washing solution and then subjected to annealing, by taking them at 75 °C for 5 min and then slowly cooling to room temperature.

The UV measurements were performed on a JASCO V-550 UV-vis spectrophotometer. A quartz cuvette with a path length of 1 cm was used. The UV quantification of the ligands was determined by measuring the absorbance relative to the λ_max_ characteristic of each ligand and referring it to the corresponding calibration curves. The errors associated with the % of bound ligand were within ±2%.

### 4.4. Circular Dichroism

CD spectra were recorded in a quartz cuvette with a path length of 1 cm, on a Jasco J-715 spectropolarimeter equipped with a Peltier-type temperature control system (model PTC-348WI). The spectra were recorded at 20 °C in the range from 240–600 nm, with 2 s response, 200 nm/min scanning speed and 2.0 nm bandwidth, and were corrected by the subtraction of the background scan with buffer. All the spectra were averaged over three scans. The oligonucleotides d[(TTAGGG)_4_TT] (tel_26_), d(TGGGGAGGGTGGGGAGGGTGGGGAAGGTGGGGA) (c-myc) and d(CGCGAATTCGCGTTTCGCGAATTCGCG) (ds_27_) were purchased from Biomers as HPLC-purified compounds with a purity of >99%. The oligonucleotides were dissolved in a 5 mM KCl, 5 mM phosphate buffer, 5% DMSO (pH 7) or 0.5 mM KCl, 0.5 mM phosphate buffer, 5% DMSO (pH 7), thus, obtaining 2 μM solutions, which were then annealed by heating at 95 °C for 5 min, followed by slow cooling to room temperature. CD titrations were obtained by adding increasing amounts of the ligands (up to 10 molar equivalents, corresponding to a 20 μM solution in ligand) to tel_26_ and c-myc G4s or ds_27_ duplex. For the CD-melting experiments, the ellipticity was recorded at 290, 262 and 251 nm for tel_26_, c-myc and ds_27_ systems, respectively, with a temperature scan rate of 0.5 °C/min, in the range from 10–95 °C.

### 4.5. Fluorescence Spectroscopy

Fluorescence spectra were recorded at 20 °C on HORIBA (Bensheim, Germany) JobinYvon Inc. FluoroMax^®^-4 spectrofluorometer equipped with F-3004 Sample Heater/Cooler Peltier Thermocouple Drive, by using a quartz cuvette with a 1 cm path length. For the fluorescence titration experiments with Bulbocapnine, Chelidonine and Ibogaine, excitation wavelengths of 307, 289 and 293 nm were used, respectively. The spectra were registered in the range from 315–600, 295–550 and 300–550 nm for Bulbocapnine, Chelidonine and Ibogaine, respectively. Titrations were carried out at a fixed concentration (2.0 μM) of ligand. Increasing amounts of tel_26_ and c-myc G4s or ds_27_ duplex (up to 10 μM concentration) were added from 120 μM annealed stock solutions of each DNA sample dissolved in a 5 mM KCl, 5 mM phosphate buffer, 5% DMSO (pH 7). After each addition, the system was allowed equilibrating 10 min before recording the spectra.

The fraction of bound molecules was calculated from the fluorescence intensity at 460, 330 and 356 nm for Bulbocapnine, Chelidonine and Ibogaine, and reported in a graph as a function of the DNA concentration.

The fraction of the bound ligand was determined using the equation:

α = (Y − Y_0_)/(Y_b_ − Y_0_) (1)

where Y, Y_0_ and Y_b_ are the values of fluorescence emission intensity at the maximum at each titrant concentration, at the initial and final state of the titration, respectively. The points were fitted with an independent and equivalent-sites model using the Origin 8.0 program.

The equation of the independent and equivalent-sites model is as follows:

(2) $$\alpha =\left(\frac{1}{2{\left[L\right]}_{0}}\right)\left\{\left({\left[L\right]}_{0}+n\left[\mathrm{DNA}\right]+\frac{1}{K_{b}}\right)-\sqrt{{\left({\left[L\right]}_{0}+n\left[\mathrm{DNA}\right]+\frac{1}{K_{b}}\right)}^{2}-4{\left[L\right]}_{0}n\left[\mathrm{DNA}\right]}\right\}$$

where α is the mole fraction of ligand in the bound form, [L]_0_ is the total ligand concentration, [DNA] is the added DNA concentration, n is the number of the equivalent and independent sites on the DNA structure and *K*_b_ is the binding constant [44].

### 4.6. Molecular Dynamics Simulations

Docking complexes were solvated in a rectangular box of TIP3P-type water molecules [72] buffering 10 Å from the macromolecule surface. The total charge was neutralized by K^+^ ions. The OL15 force field was used for DNA [73,74], while the GAFF2 force field was used for small molecules [75]. The ligands’ partial charges were computed at the am1-bcc level. The Amber18 program was used to run MD simulations [76]. In agreement with previous works [77,78,79], the following protocol was implemented herein to generate robust MD trajectories: (i) the solvent was energy minimized for 500 steps with the steepest descent algorithm (SD) followed by 2500 steps with the conjugate gradient algorithm (CG); (ii) the solvated solute was energy minimized for 1000 steps with the SD followed by 5000 steps with the CG; (iii) heating from 0 to 300 K was achieved by the Langevin thermostat at constant volume for 1 ns; iv) system density was equilibrated for 1 ns using the Berendsen barostat at constant pressure [80]; (v) the system was preliminarily relaxed for 50 ns at constant pressure; vi) final production of MD trajectories was carried out for 500 ns on each system at constant pressure without positional restraints. Time step was 2 fs in all MD simulations, which were run using the GPU version of pmemd. Analysis of MD trajectories was carried out with the CPPTRAJ program [81], while the ligands’ theoretical affinity was computed with the MMPBSA.py program [45].

### 4.7. Biological Assays

#### 4.7.1. Cells and Culture Conditions

Human fibroblasts (BJ-hTERT) were obtained by infecting primary BJ cells with a retrovirus carrying hTERT (Addgene plasmid #1773) resulting in a telomerase immortalized cell line, while BJ-EHLT were derived from the transformation of the same BJ cells with an hTERT and SV40 early region resulting in p53 and pRB silencing [82]. The human breast cancer cell line (MDA-MB-231) was purchased from ATCC. BJ-hTERT, BJ-EHLT and MDA-MB-231 were grown in Dulbecco’s Modified Eagle Medium (D-MEM, Invitrogen Carlsbad, CA, USA) supplemented with 10% Fetal Bovine Serum (FBS), 2 mM l-glutamin and antibiotics at 37 °C in a 5% CO_2_-95% air atmosphere. Stable TRF2-overexpressing cells (pBabe-puromycTRF2) and the control counterpart (pBabe-puro-Empty) [83] were obtained by infecting the cells with amphotropic retroviruses generated into Phoenix packaging cells transfected with retroviral vectors, using the JetPEI reagent (Polyplus, New York, NY, USA), according to the manufacturer’s instructions.

#### 4.7.2. Viability Assay (Crystal Violet)

BJ-EHLT and BJ-hTERT cells were seeded in a 24-well plate at a density of 5 × 10^4^ and 10 × 10^4^ for well, respectively. After 24 h, cells were treated with the compounds at different doses for 72 h. Then, cells were washed twice in 1× phosphate-buffered saline (1× PBS) and fixed with 4% formaldehyde for 15 min at room temperature (RT). After washing, 500 μL of crystal violet staining solution (Sigma-Aldrich, St. Louis, MO, USA) was added to each well and incubated for 30 min at RT. Finally, the plates were rinsed twice with water, air-dried, and cell pellets were dissolved in 250 μL of acetic acid, 10% aqueous solution. 100 μL of each sample were transferred to a 96-well plate and the optical density was measured at 570 nm (OD570) with an ELISA reader (Thermo Scientific, Waltham, MA, USA). The average absorbance in each condition was used to calculate the viability expressed as a percentage of treated vs. untreated conditions. The half-maximal viability inhibitory concentration (IC50) was calculated by CalcuSyn Version 2.1.

#### 4.7.3. Immunofluorescence (IF) and Fluorescence in situ Hybridization (FISH) Assays

For γH2AX fluorescent signal analysis, cells were fixed in 4% formaldehyde in 1× PBS for 15 min at RT and then permeabilized by treatment with 0.5% Triton X-100, 0.1% Na-Citrate (1× PBS) for 5 min at RT. Cells were blocked for 1 h in 3% BSA, 0.1% Tween-20 (1× PBS) and washed twice in 1× PBS. For immunolabeling, cells were incubated with mouse antibody mAb anti-phospho-Histone H2AX (Ser139) (Millipore, Bedford, MA, USA) primary antibody in 3% BSA, 0.1% Tween-20 (1× PBS) for 1 h at RT. Then, cells were washed in 0.3% BSA, 0.1% Tween-20 (1× PBS) and incubated for 1 h with secondary antibody Anti-Mouse IgG (H+L), F(ab’)2 Fragment (Alexa Fluor 488 Conjugate). Nuclei were stained with 4′,6-diamidino-2-phenylindole (DAPI, Sigma-Aldrich, St. Louis, MO, USA). The signal of approximately 100 nuclei for each condition was analyzed.

For immunofluorescence combined with DNA FISH, cells were fixed in 4% formaldehyde (1× PBS) followed by permeabilization with 0.1% Triton X-100 in (1× PBS) for 7 min at (RT). Cells were blocked for 1 h in 3% BSA (1× PBS) and incubated overnight with an anti-phospho-Histone H2AX antibody. After incubation with a secondary antibody, cells were fixed in 4% formaldehyde (1× PBS) and subjected to standard telomere DNA FISH. For quantitative analysis of γH2AX positivity, 300 cells on triplicate slides were analyzed. For TIFs quantification, 30 γH2AX-positive cells were scored at 63× magnification. Nuclei showing at least three colocalizations of TelC-Cy3 telomeric probe (Panagene, Daejeon, Korea) and γH2AX were considered as TIF-positive. Fluorescence signals were recorded by using a ZEISS LSM 880 confocal laser-scanning microscope by Carl Zeiss Ltd. (Oberkochen, Germany) Images were elaborated by ZEN Black 2.3 SP1 and γH2AX signal intensity was quantified using ImageJ 1.53e.

#### 4.7.4. Clonogenic Assay

MDA-MB-231 cells were seeded in a 6-well plate at the clonogenic density of 500 cells/well and after 24 h cells were treated with DMSO (negative control) or the indicated doses of Chelidonine and Rotenone. After 24 h, fresh medium was replaced in each well and cells were allowed to grow for 10 days to form colonies. Then, cells were stained with 2% methylene blue in 50% ethanol and the number of colonies was counted. Surviving fractions were calculated as the ratio of the treated vs. negative control. IC50 values were calculated by CalcuSyn Version 2.1.

#### 4.7.5. Western Blot

For Western blot analysis, cells were collected and lysed in a proper buffer (50 mM Tris-HCl pH 7.5, 5 mM EDTA, 250 mM NaCl, 0.1% Triton) completed with inhibitors of protease (ThermoScientific, A32953) and phosphatase (ThermoScientific, 88667). Total proteins were fractionated by SDS-polyacrylamide gel electrophoresis and transferred to a nitrocellulose membrane (Amersham, Arlington Heights, IL, USA). Membranes were probed with the following primary antibodies: mouse mAb anti-TRF2 (clone 4A794, Millipore, Billerica, MA, USA) and mouse mAb anti-β-actin (clone AC-15, Sigma-Aldrich, St. Louis, MO, USA).

#### 4.7.6. Statistical Analysis

The experiments were repeated three times and the obtained results are presented as mean ± standard deviation (S.D.). Statistical analysis was assessed by using the Student’s t-test for unpaired data, and the values of *p* < 0.05 were considered statistically significant. Data analysis was performed with GraphPad Prism 6 (San Diego, CA, USA).

## Figures and Tables

**Table 1:** Summary of the binding data obtained for the 28 natural compounds by the G4-CPG assay and selectivity indexes calculated as the ratio between the percentages of ligand bound to the indicated G4- and duplex-functionalized supports. Bound ligand was calculated as a difference from the unbound ligand, recovered with 50 mM KCl, 10% DMSO, 10% CH_3_CH_2_OH washing solution, and is expressed as % of the amount initially loaded on each support. The errors associated with the % are within ±2%.

	Percentage of Bound Ligand *				Selectivity Index	
	Nude CPG	CPG-tel_26_	CPG-c-myc	CPG-ds_27_	CPG-tel_26_ vs. CPG-ds_27_	CPG-c-myc vs. CPG-ds_27_
20-OH-Ecdysone	●	●	●	●	0	>2
Aloin	●	●	●	●	>2	>2
Aspidospermine	●	●	●	●	2.5	3.0
Bulbocapnine	●	●	●	●	1.1	2.3
Chelidonine	●	●	●	●	1.6	0.5
Emetine	●	●	●	●	0.3	0.7
Ferruanthrone	●	●	●	●	1.0	1.6
Ferruginin A	●	●	●	●	0.9	1.5
Ferruginin B	●	●	●	●	0	0
γ,γ’-OH-Ferruginin A	●	●	●	●	0	0
Hydrastine	●	●	●	●	1.2	1.1
Ibogaine	●	●	●	●	4.0	0
Jervine	●	●	●	●	1.1	0.9
Kuwanon G	●	●	●	●	2.7	1.5
Narceine	●	●	●	●	1.3	0.3
Rotenolone	●	●	●	●	0	0
Rotenone	●	●	●	●	1.4	0.6
Veratrine	●	●	●	●	>2	0
Vindoline	●	●	●	●	1.0	0.1
Vismione B	●	●	●	●	0	0
Vomicine	●	●	●	●	3.0	2.3
Yohimbine	●	●	●	●	>10	0

* ● = Percentage of bound ligand between 0% and 15%. ● = Percentage of bound ligand between 16% and 40%. ● = Percentage of bound ligand > 40%.

**Table 2:** Melting temperatures (T_m_) of tel_26_ and c-myc G4s or ds_27_ duplex in the absence or presence of the ligands (10 molar equivalents) as measured by CD-melting experiments.

	tel_26_ *		c-myc **		ds_27_ *	
	T_m_ (°C) (±1)	ΔT_m_ (°C)	T_m_ (°C) (±1)	ΔT_m_ (°C)	T_m_ (°C) (±1)	ΔT_m_ (°C)
**No ligand**	40		45		75	
**Bulbocapnine**	41	+1	54	+9	74	−1
**Chelidonine**	44	+4	58	+13	74	−1
**Ibogaine**	42	+2	50	+5	75	0
**Rotenone**	40	0	49	+4	75	0
**Vomicine**	40	0	50	+5	72	−3

* In 5 mM KCl, 5 mM phosphate buffer, 5% DMSO (pH 7), ** In 0.5 mM KCl, 0.5 mM phosphate buffer, 5% DMSO (pH 7).

**Table 3:** Binding constants (*K*_b_) obtained by fitting of fluorescence data for DNA/Bulbocapnine and DNA/Ibogaine systems by using an independent and equivalent-sites model [44].

	*K*_b_ (M^−1^)		
	tel_26_	c-myc	ds_27_
**Bulbocapnine**	1.0 ( ± 0.4) × 10^6^	1.1 ( ± 0.4) × 10^6^	1.2 ( ± 0.6) × 10^6^
**Ibogaine**	4.1 ( ± 0.8) × 10^5^	3.9 ( ± 0.7) × 10^5^	3.7 ( ± 0.7) × 10^5^

**Table 4:** Theoretical affinity of selected ligands to tel_26_ and c-myc G4 structures calculated along MD trajectories.

Compound	tel_26_ (MM-GBSA) kcal/mol ± SEM	c-myc (MM-GBSA) kcal/mol ± SEM
**Bulbocapnine**	−16.85 ± 0.43	−38.57 ± 0.35
**Chelidonine**	−17.49 ± 0.31	−25.07 ± 0.42
**Ibogaine**	−23.92 ± 0.34	−21.56 ± 0.71
**Rotenone**	−4.47 ± 0.73	−9.47 ± 0.42
**Vomicine**	−20.52 ± 0.33	−12.81 ± 0.49

## Data Availability

Not applicable.

## Supplementary Materials

The following are available online at https://www.mdpi.com/article/10.3390/pharmaceutics13101611/s1, Table S1: Chemical structures, features and natural sources of the 28 natural compounds here investigated, Figure S1: CD spectra of 2 μM solutions of tel_26_ G4 in 5 mM KCl, 5 mM phosphate buffer, 5% DMSO (pH 7) in the presence of increasing amounts (up to 10 equivalents) of (A) Bulbocapnine, (B) Chelidonine, (C) Ibogaine, (D) Rotenone and (E) Vomicine, Figure S2: CD spectra of 2 μM solutions of c-myc G4 in 5 mM KCl, 5 mM phosphate buffer, 5% DMSO (pH 7) in the presence of increasing amounts (up to 10 equivalents) of (A) Bulbocapnine, (B) Chelidonine, (C) Ibogaine, (D) Rotenone and (E) Vomicine, Figure S3: CD spectra of 2 μM solutions of ds_27_ duplex in 5 mM KCl, 5 mM phosphate buffer, 5% DMSO (pH 7) in the presence of increasing amounts (up to 10 equivalents) of (A) Bulbocapnine, (B) Chelidonine, (C) Ibogaine, (D) Rotenone and (E) Vomicine, Figure S4: CD spectra of solutions (from 2 to 20 µM) of (A) Bulbocapnine, (B) Chelidonine, (C) Ibogaine, (D) Rotenone and (E) Vomicine in 5 mM KCl, 5 mM phosphate buffer, 5% DMSO (pH 7), Figure S5: CD spectra (after ligand contribution subtraction) of 2 μM solutions of tel_26_ G4 in 5 mM KCl, 5 mM phosphate buffer, 5% DMSO (pH 7) with increasing amounts (up to 10 molar equivalents) of (A) Bulbocapnine, (B) Chelidonine, (C) Ibogaine, (D) Rotenone and (E) Vomicine, Figure S6: CD spectra (after ligand contribution subtraction) of 2 μM solutions of c-myc G4 in 5 mM KCl, 5 mM phosphate buffer, 5% DMSO (pH 7) with increasing amounts (up to 10 molar equivalents) of (A) Bulbocapnine, (B) Chelidonine, (C) Ibogaine, (D) Rotenone and (E) Vomicine, Figure S7: CD spectra (after ligand contribution subtraction) of 2 μM solutions of ds_27_ duplex in 5 mM KCl, 5 mM phosphate buffer, 5% DMSO (pH 7) with increasing amounts (up to 10 molar equivalents) of (A) Bulbocapnine, (B) Chelidonine, (C) Ibogaine, (D) Rotenone and (E) Vomicine, Figure S8: CD-melting curves (solid lines) for (A) tel_26_/ligand, (B) c-myc/ligand and (C) ds_27_/ligand mixtures (1:10) in 5 mM KCl, 5 mM phosphate buffer, 5% DMSO (pH 7), recorded at 290, 262 and 251 nm, respectively, and CD-melting curves (dashed lines) for (D) c-myc/ligand mixtures (1:10) in 0.5 mM KCl, 0.5 mM phosphate buffer, 5% DMSO (pH 7), recorded at 262 nm, Figure S9: CD spectra for 2 μM solutions of c-myc G4 in 5 mM KCl, 5 mM phosphate buffer, 5% DMSO (pH 7) (dashed line) or in 0.5 mM KCl, 0.5 mM phosphate buffer, 5% DMSO (pH 7) (solid line), Figure S10: Left: Fluorescence emission spectra obtained by adding increasing amounts of (A) tel_26_ G4, (B) c-myc G4 and (C) ds_27_ duplex to 2 μM solutions of Bulbocapnine. Arrows indicate the variation of fluorescence intensity on increasing DNA concentration. Right: Representative binding curves obtained by plotting the fraction of bound Bulbocapnine to (D) tel_26_ G4, (E) c-myc G4 and (F) ds_27_ duplex as a function of the DNA concentration. The black squares represent the experimental data; the red lines represent the best fit obtained using an independent and equivalent-sites model, Figure S11: Left: Fluorescence emission spectra obtained by adding increasing amounts of (A) tel_26_ G4, (B) c-myc G4 and (C) ds_27_ duplex to 2 μM solutions of Chelidonine. Arrows indicate the variation of fluorescence intensity on increasing DNA concentration. Right: Fluorescence intensity at 330 nm vs. concentration of (D) tel_26_ G4, (E) c-myc G4 and (F) ds_27_ duplex, Figure S12: Left: Fluorescence emission spectra obtained by adding increasing amounts of (A) tel_26_ G4, (B) c-myc G4 and (C) ds_27_ duplex to 2 μM solutions of Ibogaine. Arrows indicate the variation of fluorescence intensity on increasing DNA concentration. Right: Representative binding curves obtained by plotting the fraction of bound Ibogaine to (D) tel_26_ G4, (E) c-myc G4 and (F) ds_27_ duplex as a function of the DNA concentration. The black squares represent the experimental data; the red lines represent the best fit obtained using an independent and equivalent-sites model.
