# Supplementary material for: Identification of Effective Anticancer G-Quadruplex-Targeting Chemotypes through the Exploration of a High Diversity Library of Natural Compounds
Source: Pharmaceutics. 2021 Oct 3;13(10):1611. doi: 10.3390/pharmaceutics13101611 (PMC8537501; doi:10.3390/pharmaceutics13101611)
Supplement: Supplementary file 1 [file pharmaceutics-13-01611-s001.zip › pharmaceutics-1360773-supplementary.pdf]

pharmaceutics

Supplementary Material

Identification of Effective Anticancer G-Quadruplex-Targeting Chemotypes through the Exploration of a High Diversity Library of Natural Compounds

Chiara Platella [1,†], Francesca Ghirga [2,†], Pasquale Zizza [3], Luca Pompili [3], Simona Marzano [4], Bruno Pagano [4], Deborah Quaglio [2], Valeria Vergine [2], Silvia Cammarone [2], Bruno Botta [2], Annamaria Biroccio [3], Mattia Mori [5,*] and Daniela Montesarchio [1,*]

[1] Department of Chemical Sciences, University of Naples Federico II (Complesso Universitario di Monte S. Angelo), Via Cintia, 21, 80126 Napoli, Italy; chiara.platella@unina.it
[2] Department of Chemistry and Technology of Drugs, "Department of Excellence 2018−2022", Sapienza University of Rome, P.le Aldo Moro 5, 00185 Rome, Italy; francesca.ghirga@uniroma1.it (F.G.); deborah.quaglio@uniroma1.it (D.Q.); valeria.vergine@uniroma1.it (V.V.); silvia.cammarone@uniroma1.it (S.C.); bruno.botta@uniroma1.it (B.B.)
[3] Oncogenomic and Epigenetic Unit, IRCCS—Regina Elena National Cancer Institute, Via Elio Chianesi 53, 00144, Rome, Italy; pasquale.zizza@ifo.gov.it (P.Z.); luca.pompili@ifo.gov.it (L.P.); annamaria.biroccio@ifo.gov.it (A.B.)
[4] Department of Pharmacy, "Department of Excellence 2018−2022", University of Naples Federico II, via D.Montesano, 49, 80131 Napoli, Italy; simona.marzano@unina.it (S.M.); bruno.pagano@unina.it (B.P.)
[5] Department of Biotechnology, Chemistry and Pharmacy, "Department of Excellence 2018-2022", University of Siena, Via Aldo Moro 2, 53100 Siena, Italy
* Correspondence: mattia.mori@unisi.it (M.M.); daniela.montesarchio@unina.it (D.M.)
† These authors equally contributed to this work.

Citation: Platella, C.; Ghirga, F.; Zizza, P.; Pompili, L.; Marzano, S.; Pagano, B.; Vergine, V.; Cammarone, S.; Botta, B.; Biroccio, A.; et al. Identification of Effective Anticancer G-Quadruplex-Targeting Chemotypes through the Exploration of a High Diversity Library of Natural Compounds. *Pharmaceutics* **2021**, *13*, 1611. https://doi.org/10.3390/pharmaceutics13101611

Academic Editors: Jung Min Shin, Eun Sook Lee

Received: 13 August 2021
Accepted: 26 September 2021
Published: 3 October 2021

Publisher's Note: MDPI stays neutral with regard to jurisdictional claims in published maps and institutional affiliations.

Copyright: © 2021 by the authors. Licensee MDPI, Basel, Switzerland. This article is an open access article distributed under the terms and conditions of the Creative Commons Attribution (CC BY) license (http://creativecommons.org/licenses/by/4.0/).

Table S1. Chemical structures, features and natural sources of the 28 natural compounds here investigated.

Cpd	Common Name (Library Code)	Chemical Structure	M. W.	Molecular Formula	Source	Reference
		**Alkaloids**				
**1**	**Bulbocapnine· HCl (BBN196)**	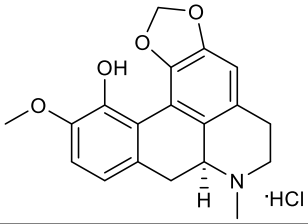	325.36 361.82 (+HCl)	$C_{19}H_{19}NO_4$· HCl	Species: *Corydalis cava* (Papaveraceae family)	[1]
**2**	**Chelidonine (BBN192)**	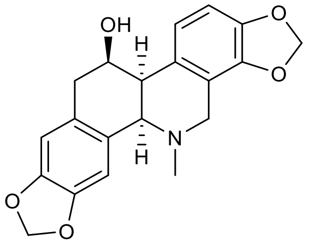	353.37	$C_{20}H_{19}NO_5$	Species: *Chelidonium majus L.* (Papaveraceae family)	[2]
**3**	**Emetine·HCl (BBN39)**	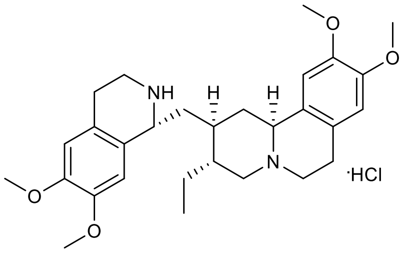	480.65 517.11 (+HCl)	$C_{29}H_{40}N_2O_4$· HCl	Species: *Psychotria ipecacuanha Stokes* (Rubiaceae family)	[3]
**4**	**Hydrastine (BBN40)**	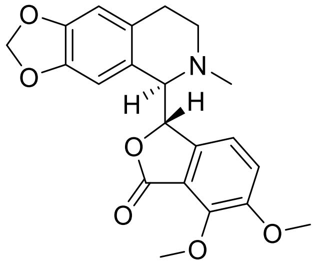	383.40	$C_{21}H_{21}NO_6$	Species: *Hydrastis canadensis L.* (Ranunculaceae family)	[4]
**5**	**Narceine (BBN254)**	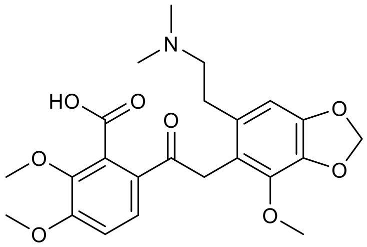	445.46	$C_{23}H_{27}NO_8$	Species: *P. somniferum L (Papaveraceae family).*	[5]
**6**	**Aspidospermine (BBN44)**	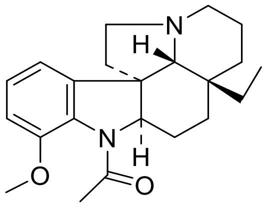	354.49	$C_{22}H_{30}N_2O_2$	Aspidosperma species: *Aspidosperma album, Aspidosperma australe, Aspidosperma exalatum, Aspidosperma peroba, Aspidosperma polyneuron,*	[6]

					Aspidosperma pyricollum, Aspidosperma pyrifolium, Aspidosperma quebracho-blanco, Aspidosperma quirandy, Aspidosperma sessiflorum, Aspidosperma rhombeosignatum (Apocynaceae family)	
**7**	**Vomicine (BBN186)**	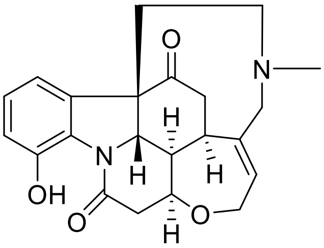	380.44	$C_{22}H_{24}N_2O_4$	Species: *Strychnos icaja* (Loganiaceae family)	[7, 8]
**8**	**Ibogaine (BBN236)**	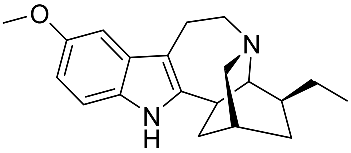	310.43	$C_{20}H_{26}N_2O$	Species: *Tabernanthe iboga* (Apocynaceae family)	[9]
**9**	**Yohimbine· HCl (BBN174)**	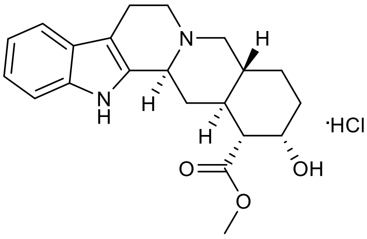	354.44 390.90 (+HCl)	$C_{21}H_{26}N_2O_3$· HCl	Species: *Aspidosperma discolor A. DC., Aspidosperma excelsum Benth, Aspidosperma eburneum F. Allem, Aspidosperma marcgravianum-Woodson, Aspidosperma oblongum A. DC* (Apocynaceae family)	[10]
**10**	**Jervine (BBN47)**	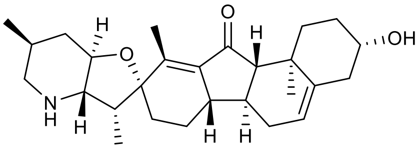	425.60	$C_{27}H_{39}NO_3$	Veratrum species: *V. album; V. dahuricum; V. lobelianum; V. nigrum; V. nigrum var. ussuriense; V. patulum; V. stenophyllum; V. taliense* (Buxaceae family)	[11]
**11**	**Solanidine (BBN209)**	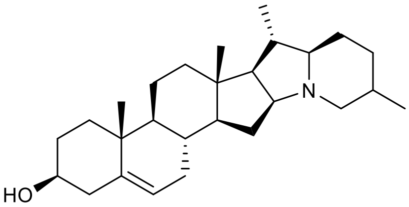	397.65	$C_{27}H_{43}NO$	Veratrum species: *V. mentzeanum; V. taliense* (Buxaceae family) Fritillaria species: *F.*	[11]

					camtschatcensis; F. tortifolia; F. ussuriensis (Liliaceae family)	
12	**Vindoline (BBN218)**	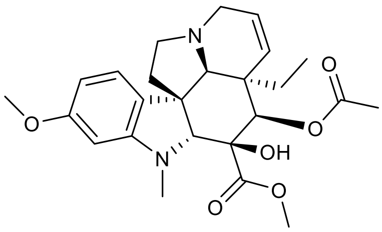	456.53	$C_{25}H_{32}N_2O_6$	Species: *Catharanthus roseus* (Apocynaceae family)	[12]
13	**Veratrine.HCl (BBN173)**	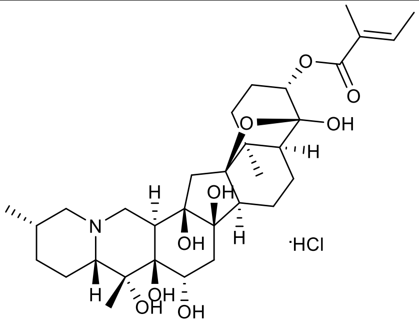	591.73 628.19 (+HCl)	$C_{32}H_{49}NO_9 \cdot HCl$	Species: *Veratrum lobelianum* (Melanthiaceae family)	[11]
			**Phenolic Compounds**			
			**Anthranoids**			
14	**Ferruginin A (BBN240)**	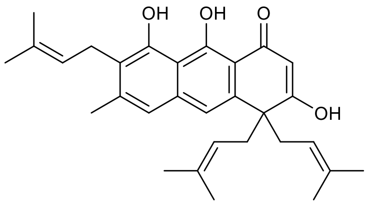	460.60	$C_{30}H_{36}O_4$	Species: *Vismia baccifera var. ferruginea* and *Vismia decipiens* (Hypericaceae family)	[13, 14]
15	**γ, γ'-OH-Ferruginin A (BBN35)**	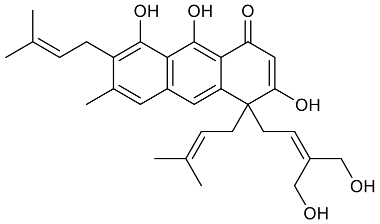	492.61	$C_{30}H_{36}O_6$	Species*:* *Vismia guaranirangae* (Hypericaceae family)	[13, 15]
16	**Ferruginin B (BBN161)**	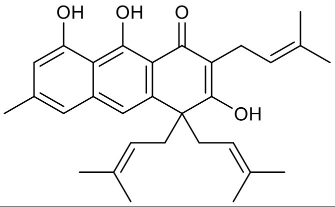	460.61	$C_{30}H_{36}O_4$	Species: *Vismia baccifera var. ferruginea* and *Vismia decipiens* (Hypericaceae family)	[13, 14]
17	**Ferruanthrone (BBN257)**	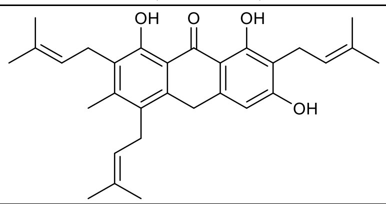	460.61	$C_{30}H_{36}O_4$	Species: *Vismia baccifera var. ferruginea* and *Vismia decipiens* (Hypericaceae family)	[13, 14]

**18**	**Aloin (BBN36)**	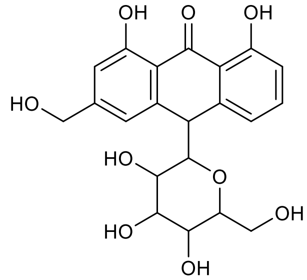	418.40	$C_{21}H_{22}O_9$	Species: *Aloe vera* (Asphodelaceae family)	[16]
**19**	**Vismione B (BBN239)**	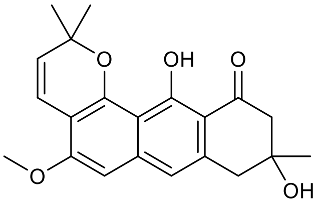	354.40	$C_{21}H_{22}O_5$	Species: *Vismia baccifera* var *dealdata* (Hypericaceae family)	[17]
			**Flavonoids**			
			**Rotenoids**			
**20**	**Rotenone (BBN255)**	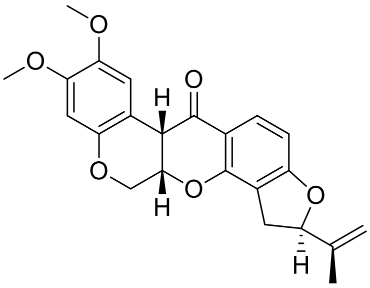	394.42	$C_{23}H_{22}O_6$	Tephrosia species: *T. cathartica Urb.* *T. sp.* *T. grandiflora Pers.* *T. maxima* *T. candida* DC. *T. subtriflora* *T. villosa Pers.* *T. wallichii Graham* *T. kolstii* *T. brachydon* *T. toxicar ú* (Sw.) *Pers.* *T. cin érea Pers.* *T. purpurea Pers.* *T. spaerospora* *T. noct flora Boj.* *T. vogelii Hook f.* (Fabaceae family)	[18, 19]
**21**	**Rotenolone (BBN75)**	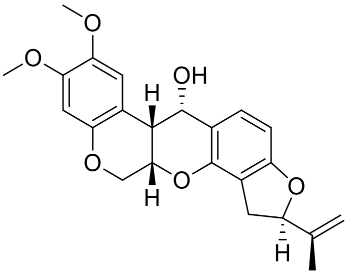	396.44	$C_{23}H_{24}O_6$	Tephrosia species: *Tephrosia vogelii* (Fabaceae family)	[19]
			**Benzophenone**			
**22**	**Clusiacitran B (BBN38)**	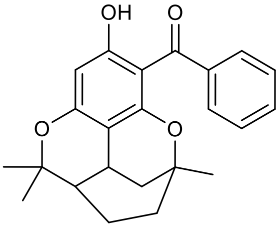	364.44	$C_{23}H_{24}O_4$	Species: *Clusia multiflora* (Clusiaceae family)	[20]
			**Steroids**			

**23**	**Diosgenin (BBN256)**	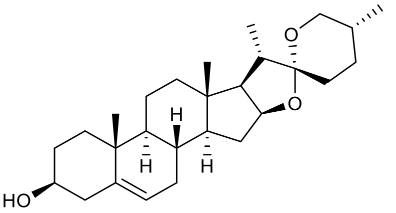	414.63	$C_{27}H_{42}O_3$	Species:*Dracaena cambodiana* and *Chlorophytum laxum* (Asparagaceae family) Species:*Balanites aegyptiaca* (Zygophyllaceae or Balanitaceae family)	[21]
**24**	**Digitonin (BBN253)**	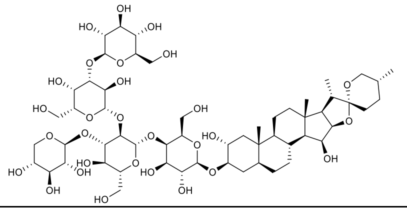	1229.32	$C_{56}H_{92}O_{29}$	Species: *Digitalis lanata* and *Digitalis purpurea* (Scrophulariaceae family)	[22]
**25**	**20-OH-Ecdysone (BBN258)**	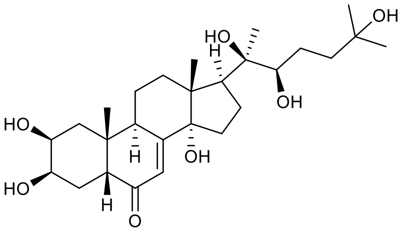	480.64	$C_{27}H_{44}O_7$	Species: *Chenopodium quinoa* Willd. (Chenopodiaceae family)	[23]
**Diels Alder type adduct**						
**26**	**Kuwanon G (BBN99)**	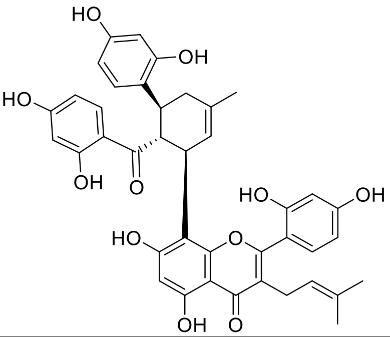	692.72	$C_{40}H_{36}O_{11}$	Species: *Morus nigra* (Moraceae family)	[24]
**Dibenzofuran**						
**27**	**Usnic acid (BBN66)**	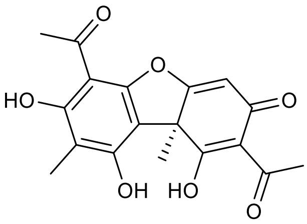	344.32	$C_{18}H_{16}O_7$	Species: *Ramalina hierensis* (Ramalinaceae family)	[25]
**Cyanogenic glycoside**						

**28**	**Amygdaline (BBN96)**	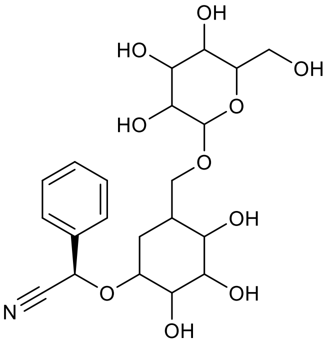	455.46	$C_{21}H_{29}NO_{10}$	Seeds of numerous members of Rosaceae family	[26]

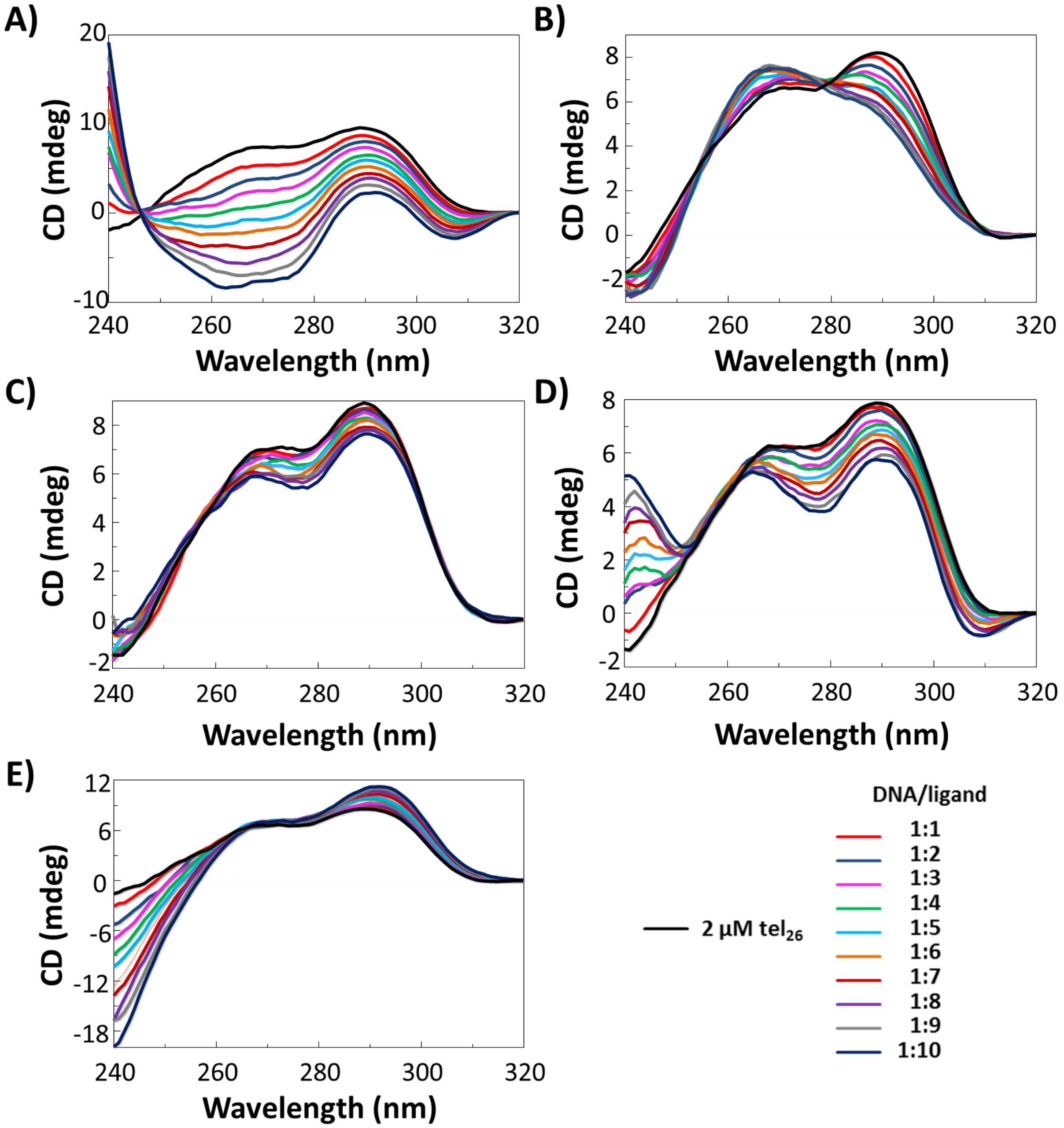

Figure S1. CD spectra of 2 µM solutions of tel_{26} G4 in 5 mM KCl, 5 mM phosphate buffer, 5% DMSO (pH 7) in the presence of increasing amounts (up to 10 equivalents) of (**A**) Bulbocapnine, (**B**) Chelidonine, (**C**) Ibogaine, (**D**) Rotenone and (**E**) Vomicine.

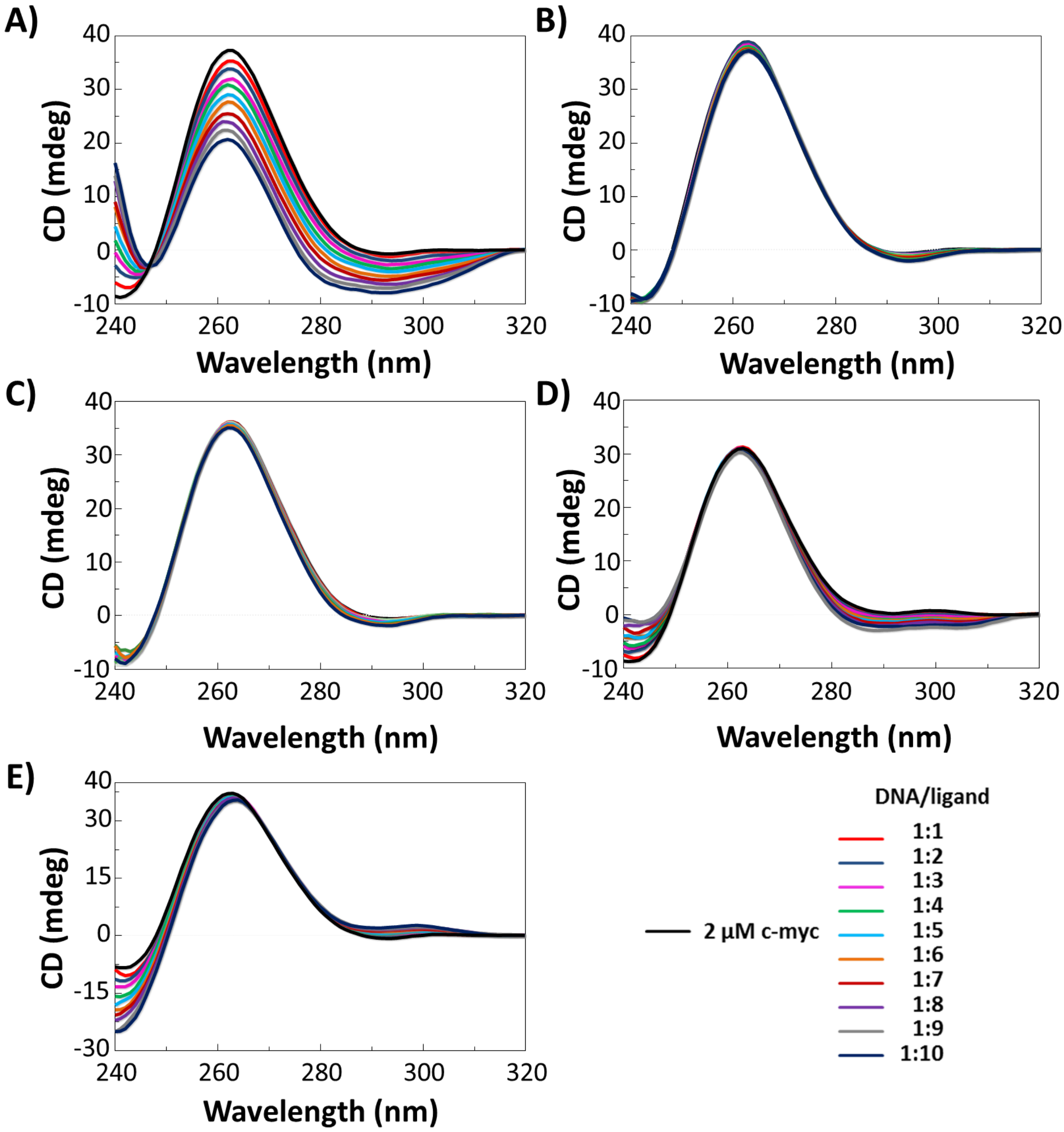

Figure S2. CD spectra of 2 µM solutions of c-myc G4 in 5 mM KCl, 5 mM phosphate buffer, 5% DMSO (pH 7) in the presence of increasing amounts (up to 10 equivalents) of (**A**) Bulbocapnine, (**B**) Chelidonine, (**C**) Ibogaine, (**D**) Rotenone and (**E**) Vomicine.

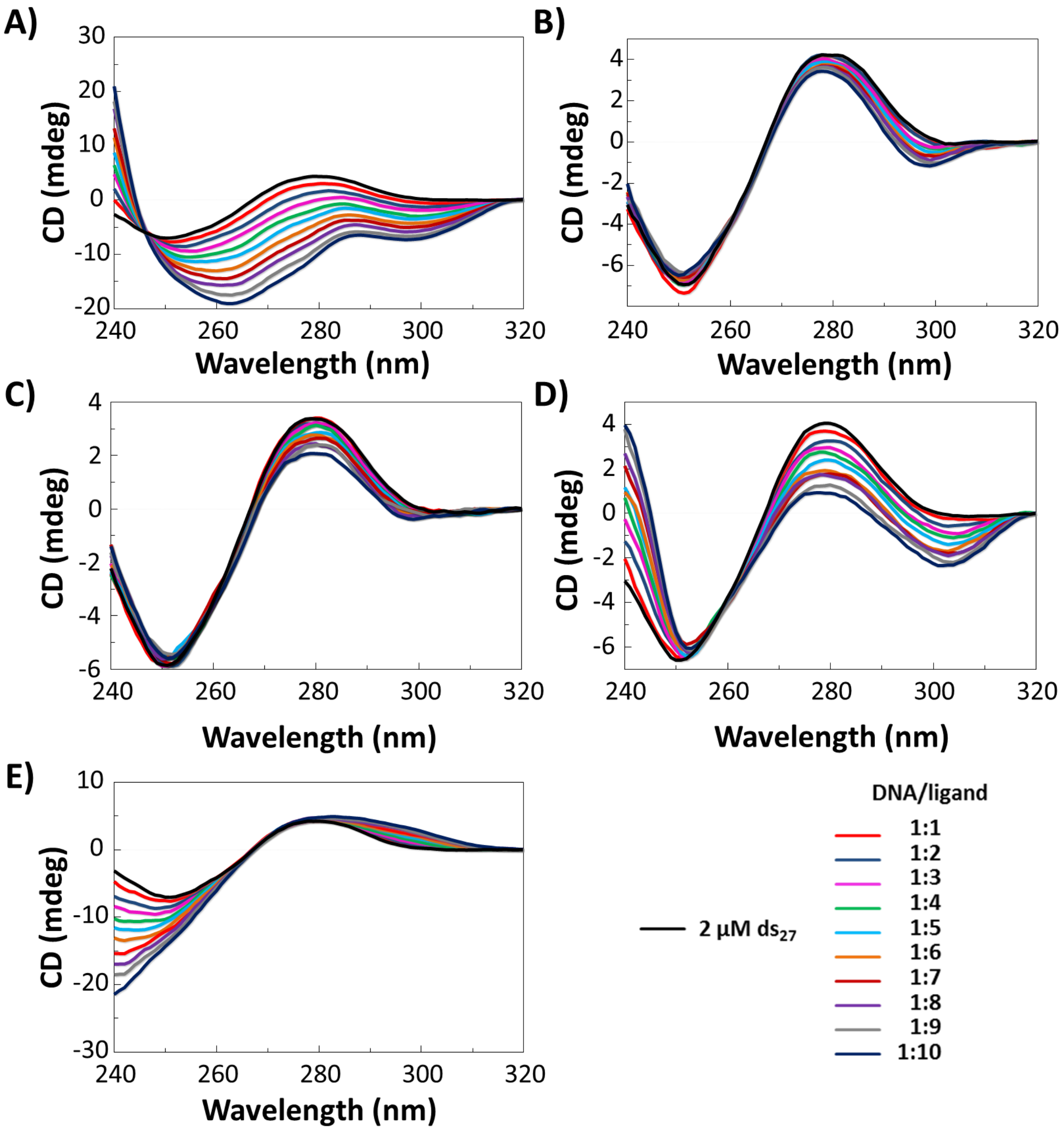

Figure S3. CD spectra of 2 μM solutions of ds27 duplex in 5 mM KCl, 5 mM phosphate buffer, 5% DMSO (pH 7) in the presence of increasing amounts (up to 10 equivalents) of (**A**) Bulbocapnine, (**B**) Chelidonine, (**C**) Ibogaine, (**D**) Rotenone and (**E**) Vomicine.

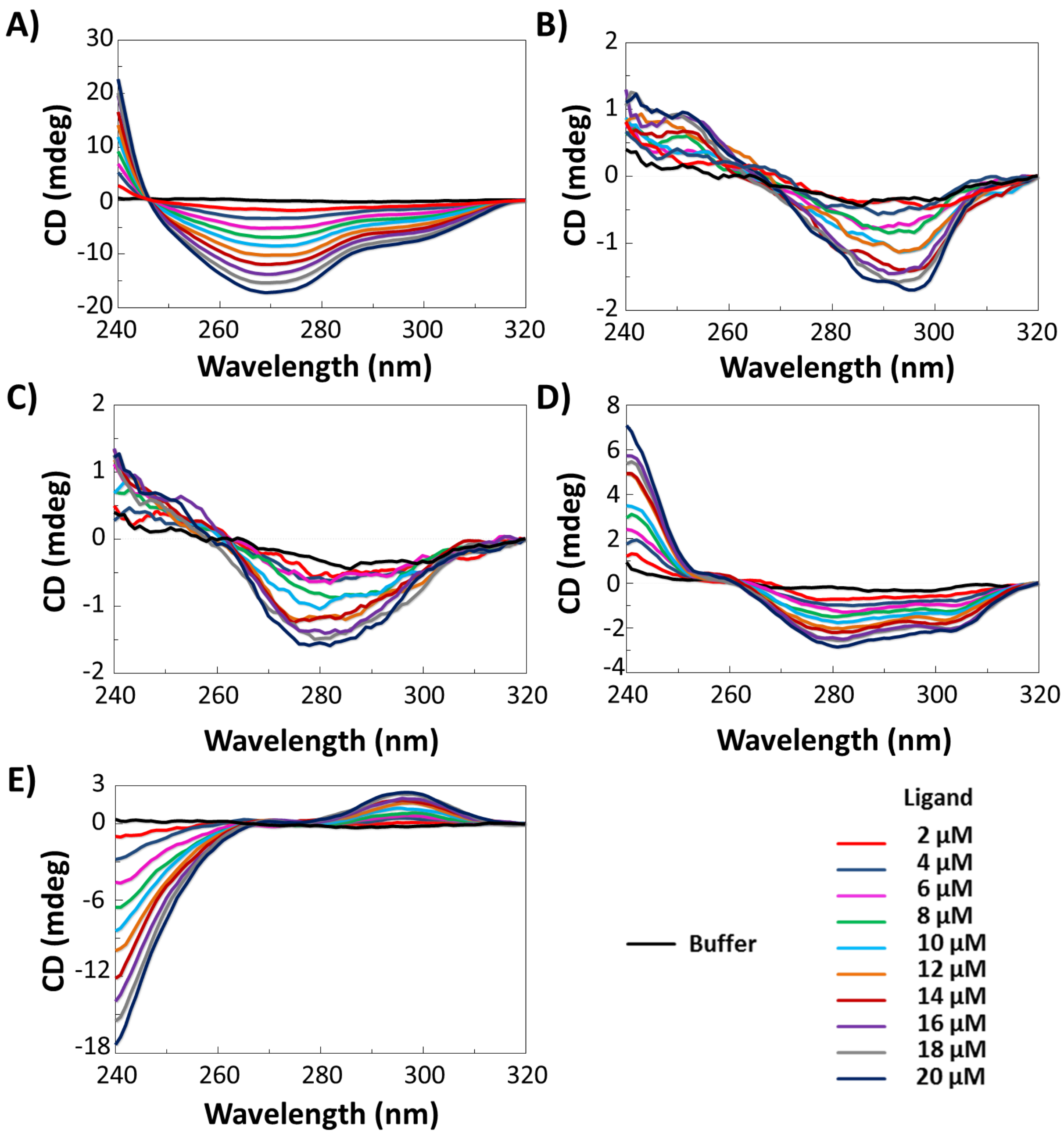

Figure S4. CD spectra of solutions (from 2 to 20 µM) of (**A**) Bulbocapnine, (**B**) Chelidonine, (**C**) Ibogaine, (**D**) Rotenone and (**E**) Vomicine in 5 mM KCl, 5 mM phosphate buffer, 5% DMSO (pH 7).

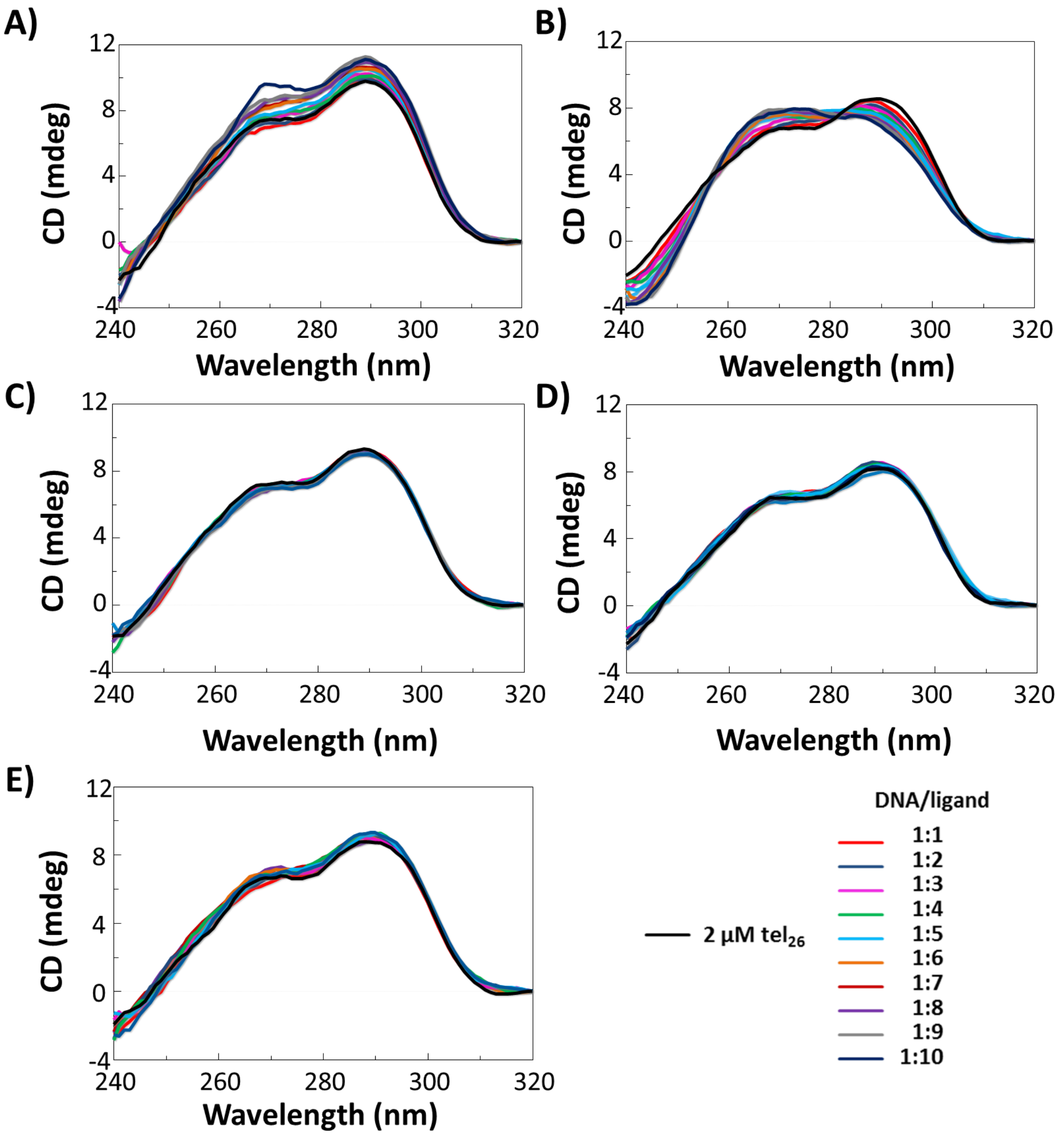

Figure S5. CD spectra (after ligand contribution subtraction) of 2 µM solutions of tel$_{26}$ G4 in 5 mM KCl, 5 mM phosphate buffer, 5% DMSO (pH 7) with increasing amounts (up to 10 molar equivalents) of (**A**) Bulbocapnine, (**B**) Chelidonine, (**C**) Ibogaine, (**D**) Rotenone and (**E**) Vomicine.

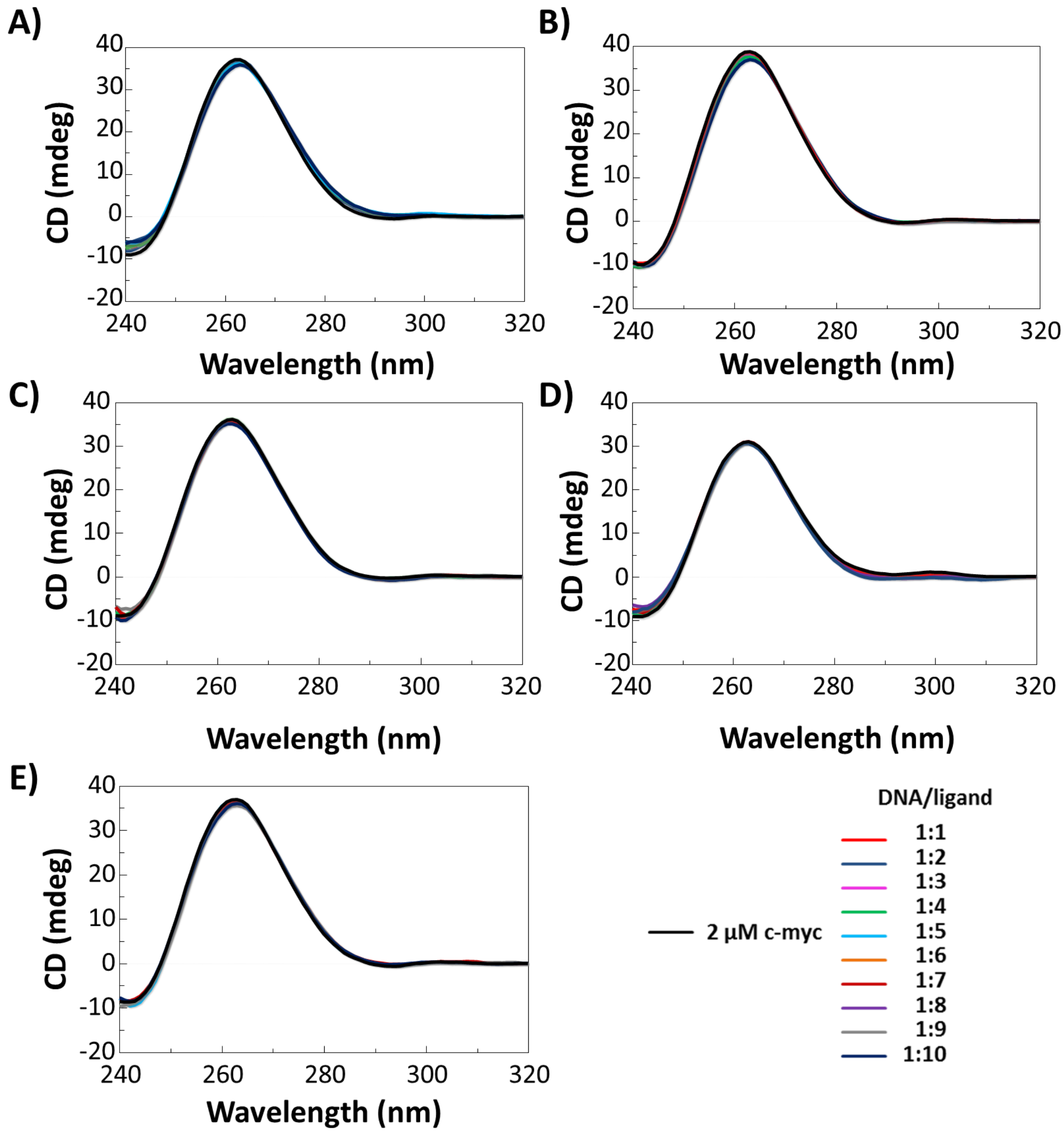

Figure S6. CD spectra (after ligand contribution subtraction) of 2 µM solutions of c-myc G4 in 5 mM KCl, 5 mM phosphate buffer, 5% DMSO (pH 7) with increasing amounts (up to 10 molar equivalents) of (**A**) Bulbocapnine, (**B**) Chelidonine, (**C**) Ibogaine, (**D**) Rotenone and (**E**) Vomicine.

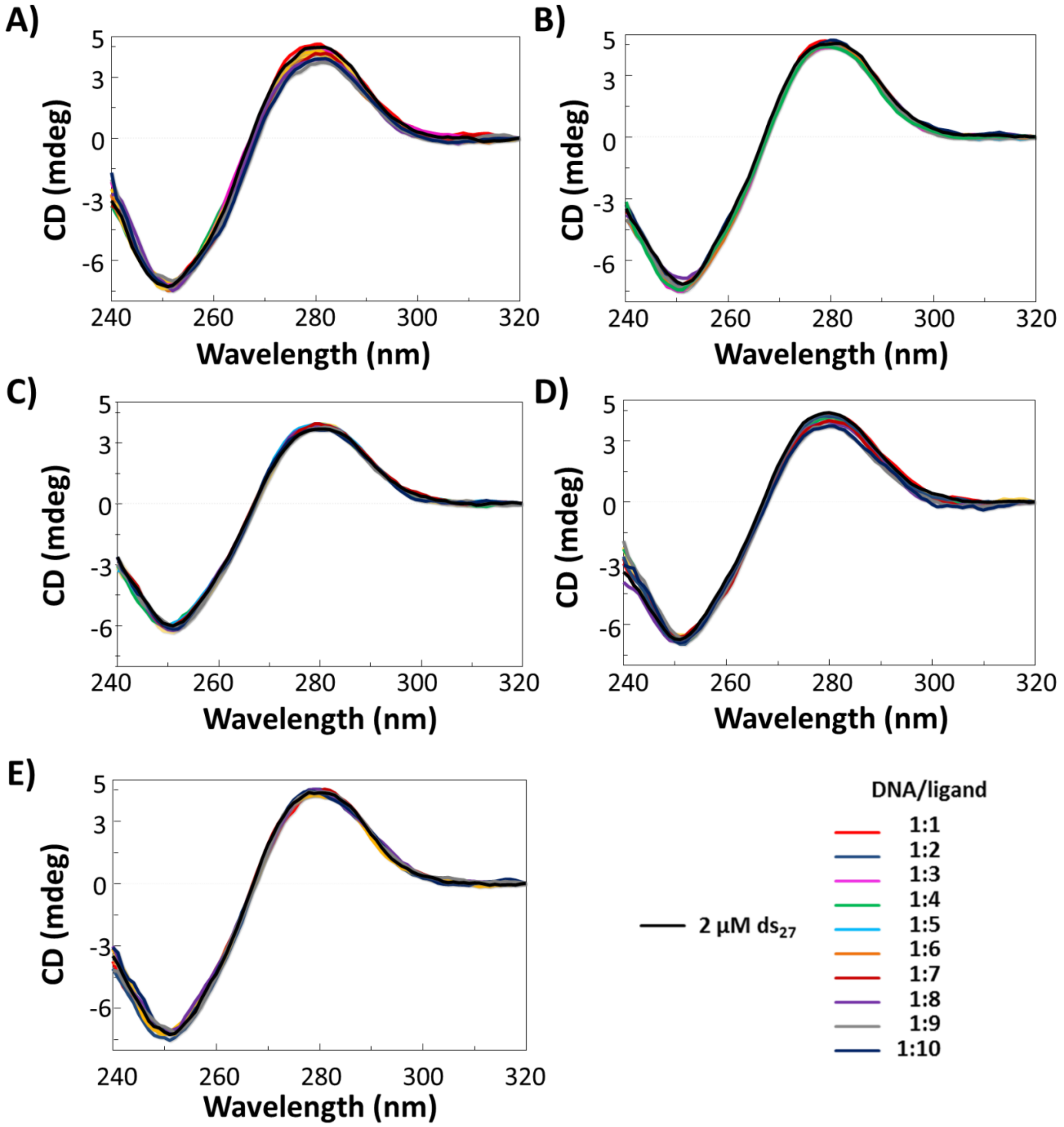

Figure S7. CD spectra (after ligand contribution subtraction) of 2 µM solutions of ds_{27} duplex in 5 mM KCl, 5 mM phosphate buffer, 5% DMSO (pH 7) with increasing amounts (up to 10 molar equivalents) of (**A**) Bulbocapnine, (**B**) Chelidonine, (**C**) Ibogaine, (**D**) Rotenone and (**E**) Vomicine.

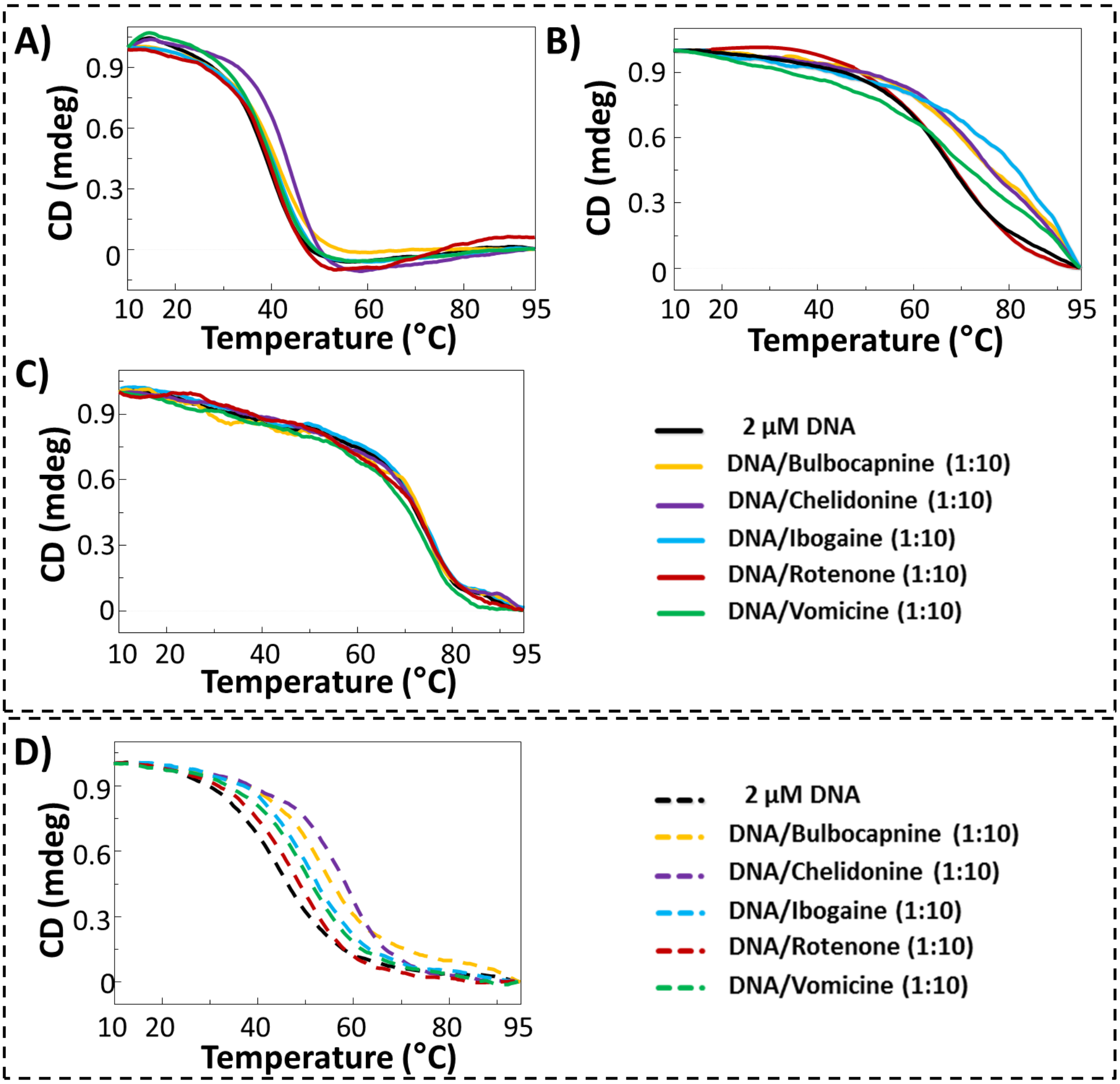

Figure S8. CD-melting curves (solid lines) for (**A**) tel26/ligand, (**B**) c-myc/ligand and (**C**) ds27/ligand mixtures (1:10) in 5 mM KCl, 5 mM phosphate buffer, 5% DMSO (pH 7), recorded at 290, 262 and 251 nm, respectively, and CD-melting curves (dashed lines) for (**D**) c-myc/ligand mixtures (1:10) in 0.5 mM KCl, 0.5 mM phosphate buffer, 5% DMSO (pH 7), recorded at 262 nm.

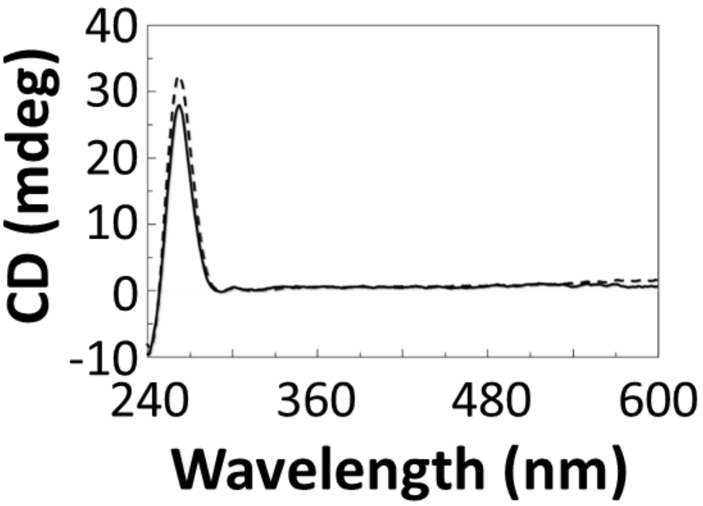

Figure S9. CD spectra for 2 μM solutions of c-myc G4 in 5 mM KCl, 5 mM phosphate buffer, 5% DMSO (pH 7) (dashed line) or in 0.5 mM KCl, 0.5 mM phosphate buffer, 5% DMSO (pH 7) (solid line).

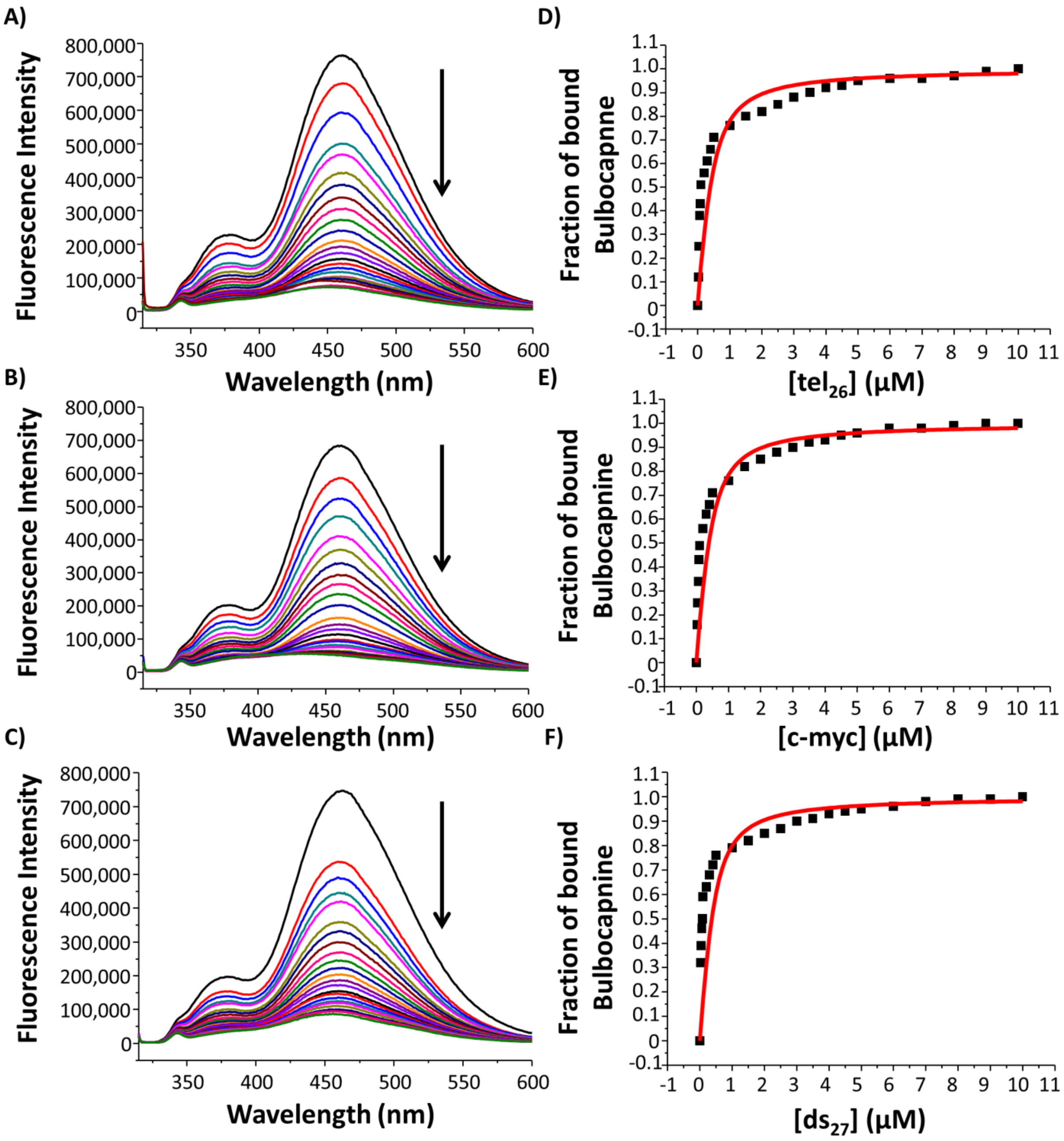

Figure S10. Left: Fluorescence emission spectra obtained by adding increasing amounts of (**A**) tel_{26} G4, (**B**) c-myc G4 and (**C**) ds_{27} duplex to 2 μM solutions of Bulbocapnine. Arrows indicate the variation of fluorescence intensity on increasing DNA concentration. Right: Representative binding curves obtained by plotting the fraction of bound Bulbocapnine to (**D**) tel_{26} G4, (**E**) c-myc G4 and (**F**) ds_{27} duplex as a function of the DNA concentration. The black squares represent the experimental data; the red lines represent the best fit obtained using an independent and equivalent-sites model.

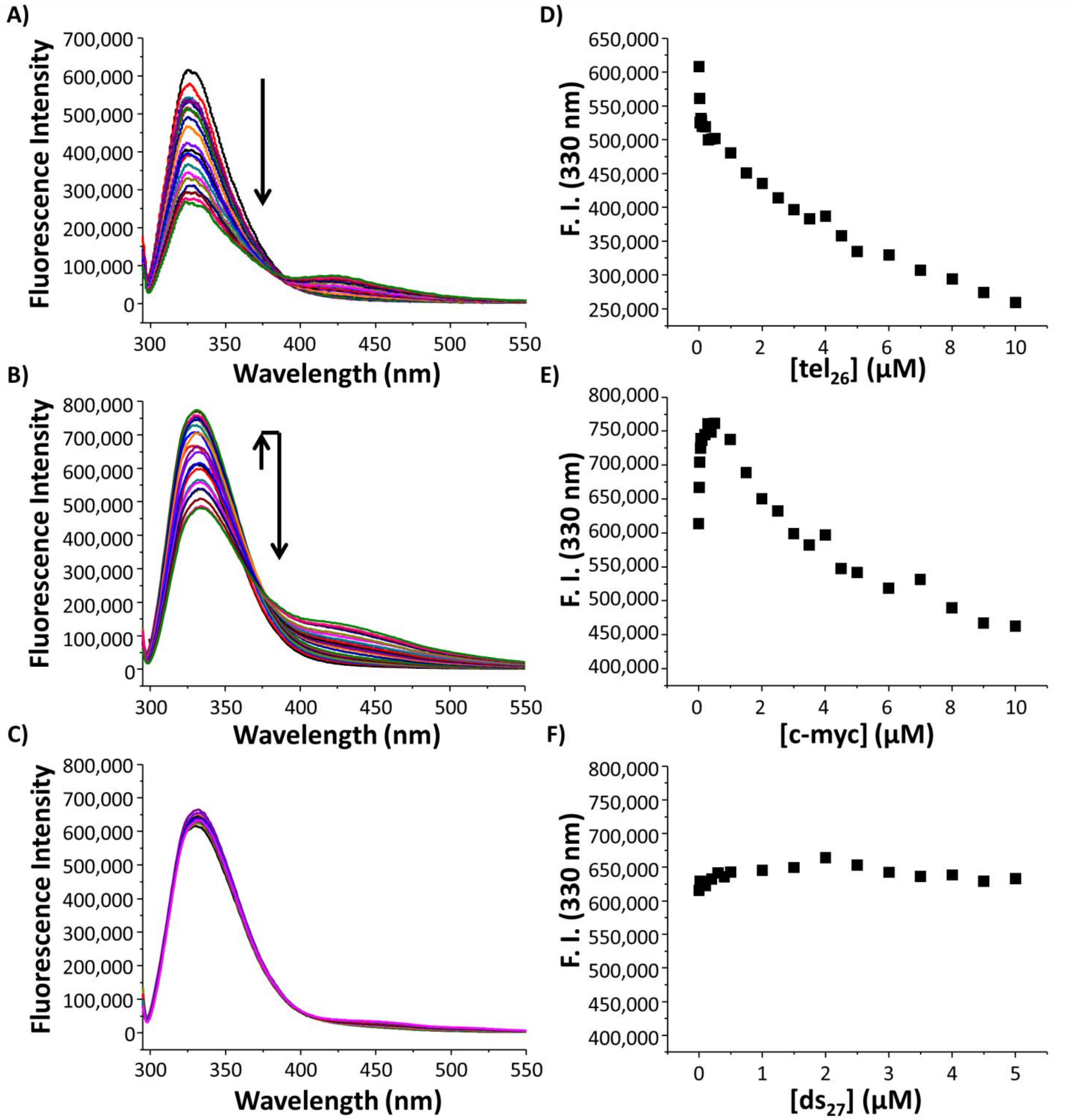

Figure S11. Left: Fluorescence emission spectra obtained by adding increasing amounts of (**A**) tel_{26} G4, (**B**) c-myc G4 and (**C**) ds_{27} duplex to 2 μM solutions of Chelidonine. Arrows indicate the variation of fluorescence intensity on increasing DNA concentration. Right: Fluorescence intensity at 330 nm vs. concentration of (**D**) tel_{26} G4, (**E**) c-myc G4 and (**F**) ds_{27} duplex.

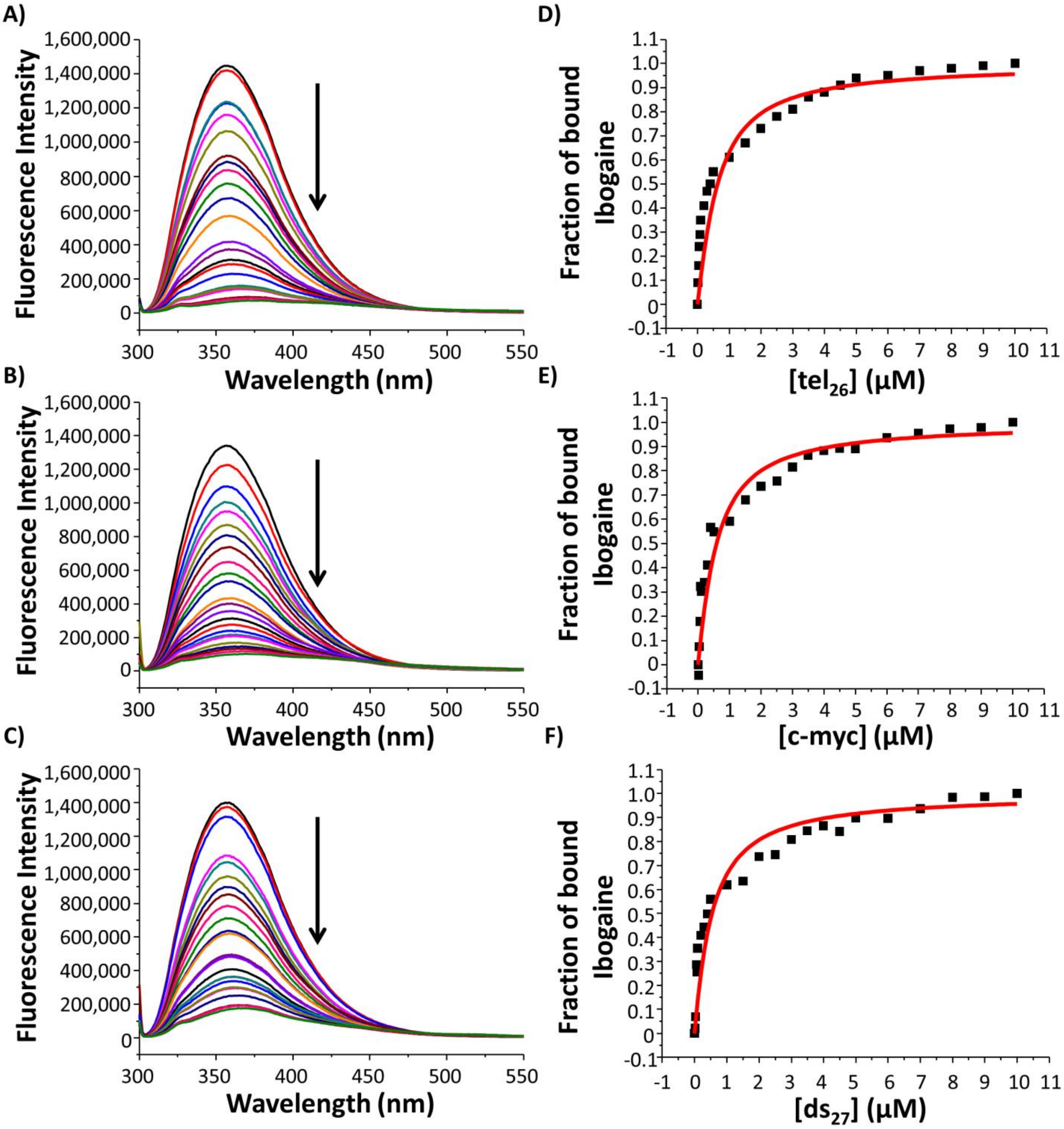

Figure S12. Left: Fluorescence emission spectra obtained by adding increasing amounts of (**A**) tel26 G4, (**B**) c-myc G4 and (**C**) ds27 duplex to 2 μM solutions of Ibogaine. Arrows indicate the variation of fluorescence intensity on increasing DNA concentration. Right: Representative binding curves obtained by plotting the fraction of bound Ibogaine to (**D**) tel26 G4, (**E**) c-myc G4 and (**F**) ds27 duplex as a function of the DNA concentration. The black squares represent the experimental data; the red lines represent the best fit obtained using an independent and equivalent-sites model.

References

1. Meyer, A.; Imming, P. Benzylisoquinoline Alkaloids from the Papaveraceae: The Heritage of Johannes Gadamer (1867–1928). *J. Nat. Prod.* **2011**, *74*, 2482–2487, doi:10.1021/np2005049.
2. Colombo, M.L. Pharmacological activities of chelidonium majusl. (papaveraceae). *Pharmacol. Res.* **1996**, *33*, 127–134, doi:10.1006/phrs.1996.0019.
3. Akinboye, E.S. Biological Activities of Emetine. *Open Nat. Prod. J.* **2011**, *4*, 8–15, doi:10.2174/1874848101104010008.
4. Brown, P.N.; Roman, M.C.; Chang, C.; Jin, C.; Kuriyedath, R.; Lidstone, S.; Ly, L.; Ma, Y.-C.; Sigmund, P.; Smith, R.; et al. Determination of Hydrastine and Berberine in Goldenseal Raw Materials, Extracts, and Dietary Supplements by High-Performance Liquid Chromatography with UV: Collaborative Study. *J. AOAC Int.* **2008**, *91*, 694–701, doi:10.1093/jaoac/91.4.694.
5. Šantavý, F. Chapter 4 Papaveraceae Alkaloids. II. In The Alkaloids: Chemistry and Physiology; Elsevier BV, 1979; Vol. 17, pp. 385–544.
6. Guimarães, H.A.; Braz-Filho, R.; Vieira, I.J.C. 1H and 13C-NMR Data of the Simplest Plumeran Indole Alkaloids Isolated from Aspidosperma Species. *Mol.* **2012**, *17*, 3025–3043, doi:10.3390/molecules17033025.
7. Monache, F.D.; Monache, G.D.; Souza, M.A.D.M.E.; Cavalcanti, M.D.S.; Chiappeta, A. ChemInform Abstract: Isopentenylindole Derivatives and Other Components of Esenbeckia leiocarpa. *Chemin-* **1990**, *21*, 435–439, doi:10.1002/chin.199005299.
8. Ohiri, F.; Verpoorte, R.; Svendsen, A. The African strychnos species and their alkaloids: A review. *J. Ethnopharmacol.* **1983**, *9*, 167–223, doi:10.1016/0378-8741(83)90032-6.
9. Alper, K.R. Chapter 1 Ibogaine: A review. *The Alkaloids* **2001**, *56*, 1–38, doi:10.1016/s0099-9598(01)56005-8.
10. Amaral, A.C.F.; Ramos, A.D.S.; Ferreira, J.L.P.; Dos Santos, A.R.; Da Cruz, J.D.; De Luna, A.V.M.; Nery, V.V.C.; De Lima, I.C.; Chaves, M.H.D.C.; Silva, J.R.D.A.; et al. LC-HRMS for the Identification of β-Carboline and Canthinone Alkaloids Isolated from Natural Sources. *Mass Spectrometry* **2017**, *6*, 187–207,, doi:10.5772/68075.
11. Li, H.-J.; Jiang, Y.; Li, P. Chemistry, bioactivity and geographical diversity of steroidal alkaloids from the Liliaceae family. *Nat. Prod. Rep.* **2006**, *23*, 735–752, doi:10.1039/b609306j.
12. Song, K.M.; Park, S.W.; Hong, W.H.; Lee, H.; Kwak, S.S.; Liu, J.R. Isolation of vindoline from Catharanthus roseus by supercritical fluid extraction. *Biotechnol. Prog.* **1992**, *8*, 583–586, doi:10.1021/bp00018a018.
13. Monache, G.D.; Gonzalez, J.; Bettolo, G. Prenylated benzophenones from Vismia decipiens. *Phytochemistry* **1980**, *19*, 2025–2028, doi:10.1016/0031-9422(80)83030-5.
14. Monache, F.; Mc Quhae, M.; Ferrari, F.; Marini-Bettolo, G. Ferruginin A and B and ferruanthrone, new triprenylated anthranoids from Vismia baccifera var. ferruginea. *Tetrahedron* **1979**, *35*, 2143–2149, doi:10.1016/0040-4020(79)87031-3.
15. Monache, F.D.; Torres, F.F.; Marini-Bettolo, G.B.; De Lima, R.A. Chemistry of Vismia Genus. Note V: γ-Hydroxy- and γ,γ′-Dihydroxy-Ferruginin A. *J. Nat. Prod.* **1980**, *43*, 487–494, doi:10.1021/np50010a009.
16. Patel, K.; Patel, D.K. Medicinal importance, pharmacological activities, and analytical aspects of aloin: A concise report. *J. Acute Dis.* **2013**, *2*, 262–269, doi:10.1016/s2221-6189(13)60141-9.
17. Monache, F.D.; Ferrari, F.; Marini-Bettolo, G.B., Maxfield, P.; et al. Vismiones from Vismia baccifera var. dealdata (H.B.K.): Chemistry and X-ray structure determination. *Gazz. Chim. Ital*. **1979**, *109*, 301–310.
18. Delfel, N.E.; Tallent, W.H.; Carlson, D.G.; Wolff, I.A. Distribution of rotenone and deguelin in Tephrosia vogelii and separation of rotenoid-rich fractions. *J. Agric. Food Chem.* **1970**, *18*, 385–390, doi:10.1021/jf60169a053.
19. Irvine, J.E.; Freyre, R.H. Source Materials for Rotenone, Occurrence of Rotenoids in Some Species of the Genus Tephrosia. *J. Agric. Food Chem.* **1959**, *7*, 106–107, doi:10.1021/jf60096a002.
20. Gonzalez, J.G.; Olivares, E.M.; Monache, F.D. Citrans and cyclols from Clusia multiflora. *Phytochemistry* **1995**, *38*, 485–489, doi:10.1016/0031-9422(94)00642-7.
21. Sobolewska, D.; Galanty, A.; Grabowska, K.; Makowska-Wąs, J.; Wróbel-Biedrawa, D.; Podolak, I. Saponins as cytotoxic agents: an update (2010–2018). Part I—steroidal saponins. *Phytochem. Rev.* **2020**, *19*, 139–189, doi:10.1007/s11101-020-09661-0.
22. Jograna, M.; Patil, D.; Kotwal, S.V., Digitalis species a potent herbal drug: A review on their pharmacognosy and pharmacological activities. *J. Curr. Pharms Res.* **2020**, *10(4)*, 3821–3831.
23. Zughdani, M.; Yusufoğlu, H.S.; Ekiz, G.; Linden, A.; Çalış, I. Ecdysteroids from the underground parts of Rhaponticum acaule (L.) DC. *Phytochemistry* **2020**, *180*, 112530, doi:10.1016/j.phytochem.2020.112530.
24. Mascarello, A.; Chiaradia-Delatorre, L.D.; Mori, M.; Terenzi, H.; Botta, B. Mycobacterium tuberculosis-Secreted Tyrosine Phosphatases as Targets Against Tuberculosis: Exploring Natural Sources in Searching for New Drugs. *Curr. Pharm. Des.* **2016**, *22*, 1561–1569, doi:10.2174/1381612822666160112130539.
25. González, A.G.; Pérez, E.M.R.; Barrera, J.B. Biologically Active Compounds from the LichenRamalina hierrensis. *Planta Medica* **1991**, *57*, A2, doi:10.1055/s-2006-960238.
26. Botelho, A.F.M.; Pierezan, F.; Soto-Blanco, B.; Melo, M.M. A review of cardiac glycosides: Structure, toxicokinetics, clinical signs, diagnosis and antineoplastic potential. *Toxicon* **2019**, *158*, 63–68, doi:10.1016/j.toxicon.2018.11.429.
